# Integrating Dynamic Red Blood Cell Distribution Width Monitoring and β-Blocker Therapy for Mortality Prediction in Intensive Care Unit Cardiomyopathy Patients: A Bayesian Multivariate Joint Model and Machine Learning Study

**DOI:** 10.3390/diagnostics15101236

**Published:** 2025-05-14

**Authors:** Si Chen, Rui Nie, Yi Wang, Haoran Guo, Yan Wang, Haixia Luan, Xiaoli Zeng, Hui Yuan

**Affiliations:** Department of Clinical Laboratory, Beijing Anzhen Hospital, Capital Medical University, Beijing 100029, China; chensi888.hi@163.com (S.C.); nierui0615@163.com (R.N.); wangyizzzccc@163.com (Y.W.); guohaoran0027@163.com (H.G.); 13801041202@163.com (Y.W.); haixialuan888@163.com (H.L.)

**Keywords:** cardiomyopathy, ICU mortality, red blood cell distribution width, β-blockers, dynamic monitoring, Bayesian multivariate joint model, machine learning

## Abstract

**Background/Objective:** Cardiomyopathy is a key cause of cardiovascular mortality in critically ill patients. Although red blood cell distribution width (RDW) is recognized as a potential prognostic biomarker, its variations during ICU admission and its interaction with treatments such as β-blockers are not well understood across different cardiomyopathy subtypes. To assess the prognostic significance of RDW dynamics and their interaction with β-blocker therapy in predicting 365-day mortality among ICU patients with dilated, hypertrophic, and restrictive cardiomyopathy, utilizing longitudinal data and advanced modeling techniques. **Methods:** A retrospective analysis was conducted on 317 cardiomyopathy patients from the MIMIC-IV database. Their RDW dynamics were assessed over their ICU stay. Cox regression (including time-dependent Cox models) and logistic regression identified independent mortality risk factors. Key predictors were identified using Least absolute shrinkage and selection operator (LASSO) regression and the Boruta algorithm. Restricted cubic splines (RCSs) were used to examine nonlinear relationships. Machine learning models were used to evaluate predictive performance, with SHapley Additive Explanations (SHAP) and tree-based feature selection identifying influential variables. Repeated-measures ANOVA was used to analyze RDW trends and β-blocker associations. A Bayesian multivariate joint model (BMJM) integrated RDW dynamics and β-blocker therapy, incorporating repeated measures and survival outcomes. **Results:** RDW was an independent predictor of 365-day mortality (HR = 1.14, 95% CI: 1.01–1.29, *p* = 0.03), alongside the systemic immune-inflammation index (SII) (HR = 1.01, 95% CI: 1.00–1.01, *p* = 0.03), whereas β-blockers significantly reduced mortality risk (HR = 0.2, 95% CI: 0.1–0.39, *p* < 0.001). Comparative analysis demonstrated that RDW exhibited greater predictive value over the aggregate index of systemic inflammation (AISI), systemic inflammation response index (SIRI), and SII. Machine learning identified logistic classification as the best predictive model (AUC = 0.811), with SHAP and tree-based selection confirming RDW and β-blockers as key predictors. A repeated-measures ANOVA revealed a significant interaction between RDW and β-blocker use (F = 6.65, *p* < 0.0001), with β-blockers lowering RDW levels. The BMJM demonstrated strong predictive performance (AUC = 0.80). The patient-specific BMJM indicated that discontinuing β-blockers increased the risk of mortality, while initiating β-blockers reduced it. **Conclusions:** This study highlights dynamic RDW monitoring and β-blocker therapy as strong predictors of 365-day mortality in ICU-admitted cardiomyopathy patients. The BMJM enables personalized risk assessment by integrating longitudinal biomarker data. These findings support RDW as a dynamic biomarker and advocate for its integration into personalized treatment strategies.

## 1. Introduction

Cardiomyopathy encompasses a group of myocardial diseases with diverse causes and complex clinical presentations, often leading to sudden cardiac death and heart failure. Globally, heart failure affects more than 64 million individuals [1]. Cardiovascular diseases (CVDs) remain the leading cause of mortality worldwide. The World Health Organization reports that cardiovascular diseases caused about 17.9 million deaths in 2019, accounting for 32% of worldwide fatalities. Cardiomyopathy contributes substantially to this burden due to its pivotal role in the development of heart failure and arrhythmia-related mortality. According to the 2023 ESC guidelines, cardiomyopathies are categorized into five types based on morphology and function: hypertrophic (HCM), dilated (DCM), arrhythmogenic right ventricular (ARVC), restrictive (RCM), and non-dilated left ventricular (NDLVC) [2]. DCM and HCM are the predominant types, comprising over 90% of cases, whereas ARVC, RCM, and NDLVC each represent 2.1% to 3.6% [3]. The prevalence of DCM is estimated at 36.5 per 100,000 population [4], while HCM affects approximately 1 in 200–500 individuals [5]. The guidelines recommend multimodal imaging for diagnosis, alongside family screening, genetic testing, and laboratory investigations [2]. Conventional treatments include ACEIs/ARBs, β-blockers, aldosterone antagonists, diuretics, inotropes, anticoagulants, devices such as pacemakers, cardiac resynchronization therapy, and heart transplantation [5,6]. Innovative treatments like gene editing, gene replacement, and allele-specific silencing present novel therapeutic opportunities [7,8,9,10].

Prior research has explored the prognostic significance of red blood cell distribution width (RDW) in heart failure (HF) patients, especially those with HF due to DCM. In DCM patients with a left ventricular ejection fraction <30%, an RDW ≥15% is linked to increased long-term mortality or the necessity for heart transplantation [11]. Elevated RDW is recognized as an independent mortality predictor in patients with coronary heart disease and DCM-related HF [12]. Elevated RDW serves as a significant prognostic marker for mortality in adult HCM patients, with higher RDW tertiles being associated with increased all-cause mortality and HCM-related mortality [13]. Systemic inflammatory markers, such as the aggregate index of systemic inflammation (AISI), the systemic inflammation response index (SIRI), and the systemic immune-inflammation index (SII), show a significant positive correlation with the incidence of HF, with higher levels of these markers associated with an increased risk of HF [14]. Moreover, a significant association has been observed between SIRI and SII levels and the risk of mortality in patients with chronic HF [15]. Furthermore, the SII has been identified as a useful risk stratification factor for mortality in patients with HCM [16]. Therefore, previous studies have primarily explored the relationships between RDW, AISI, SIRI, and SII and HF or HF caused by cardiovascular diseases. However, to our knowledge, no prior research has specifically examined the associations between these inflammatory markers and all-cause mortality in patients with distinct cardiomyopathy subtypes, including DCM, HCM, and RCM. Furthermore, the potential interaction between these biomarkers and pharmacological interventions—particularly β-blocker therapy—remains uninvestigated. This gap in knowledge highlights the need for studies that integrate biomarker dynamics with treatment strategies to better understand prognosis in cardiomyopathy.

The primary objectives of this study are twofold: (1) Cross-sectional analysis: to identify key prognostic factors associated with 365-day mortality among ICU-admitted patients diagnosed with DCM, HCM, and RCM. This objective was addressed through the application of least absolute shrinkage and selection operator (LASSO) regression, Cox proportional hazards models (including time-dependent Cox regression), logistic regression, Kaplan–Meier (KM) survival analysis, and restricted cubic spline (RCS) modeling. Machine learning algorithms were employed to construct optimal predictive models, with SHapley Additive Explanations (SHAP) and tree-based feature selection were used to identify and rank the most influential predictors. (2) Longitudinal dynamic data analysis: to characterize the temporal trajectory of RDW during ICU admission and explore its interaction with β-blocker therapy. This was achieved through the use of trend curve analysis and repeated-measures analysis of variance (ANOVA). Additionally, a Bayesian multivariate joint model (BMJM) was developed to jointly model RDW dynamics and survival outcomes, thereby enabling individualized prediction of 365-day mortality and supporting personalized treatment decision-making.

## 2. Methods

### 2.1. Data Source

In this study, we analyzed data from version 3.1 of the Medical Information Mart for Intensive Care-IV (MIMIC-IV) database [17], a comprehensive and deidentified repository of healthcare records. This dataset encompasses medical information from over 65,000 patients admitted to intensive care units (ICUs) and more than 200,000 visits to the emergency department at Beth Israel Deaconess Medical Center (Boston, MA, USA), spanning the period from 2008 to 2022 [18]. The first author, S. C., obtained access to the MIMIC-IV database after successfully completing the Collaborative Institutional Training Initiative program (certification number: 64388854). This study adhered to the principles outlined in the Declaration of Helsinki, and the requirement for informed consent was waived due to the anonymized nature of the data. Ethical approval for this study was granted by the institutional review board of Beth Israel Deaconess Medical Center.

### 2.2. Study Population

**Figure 1:**
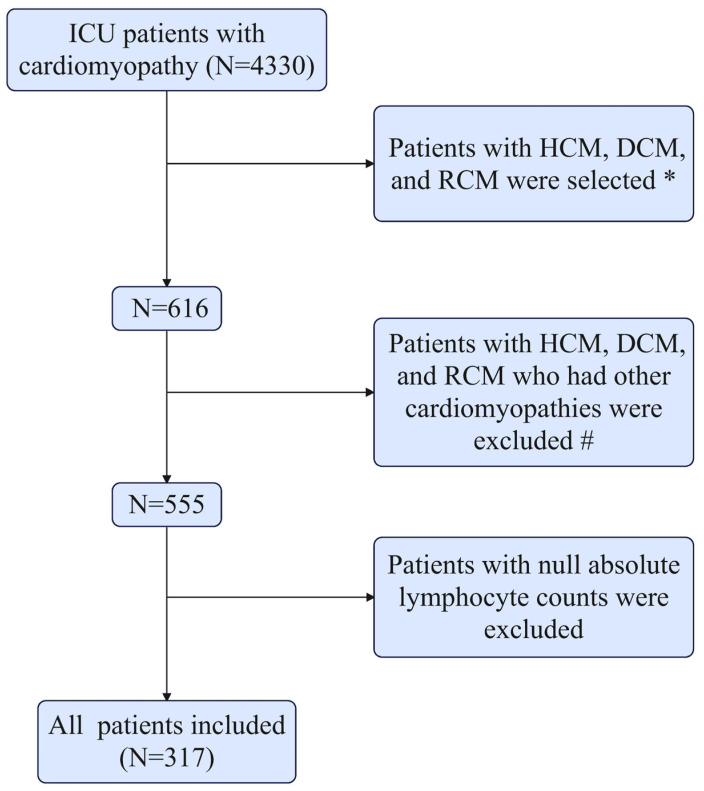
Flowchart of patient inclusion and exclusion from the MIMIC-IV database. Abbreviations: DCM: dilated cardiomyopathy; HCM: hypertrophic cardiomyopathy; RCM: restrictive cardiomyopathy; ICU: intensive care unit; MIMIC-IV: Medical Information Mart for Intensive Care-IV. * The ICD codes were ICD-9-CM: 425.1; and ICD-10-CM: I42.0, I42.1, I42.2, and I42.5. # The ICD codes were ICD-10-CM: A36.81, B33.24, I25.5, I42, I42.3, I42.6–I42.9, I43, and O90.3; and ICD-9-CM: 425.2, 425.4, 425.5, 425.7–425.9, and 674.50–674.54.

This study exclusively included patients with cardiomyopathy who were admitted to the ICU. We retrospectively identified patients from the MIMIC-IV database who had been diagnosed with DCM, HCM, or RCM based on International Classification of Diseases (ICD) codes: ICD-9 code 425.1 and ICD-10 codes I42.0, I42.1, I42.2, and I42.5. For patients with multiple ICU admissions, only data from the first ICU stay were included in the analysis. Patients were excluded if they were under 18 years of age, had incomplete medical records, or had other concurrent cardiomyopathies. Specifically, patients with HCM, DCM, or RCM who had overlapping diagnoses of other cardiomyopathy subtypes were excluded. Additionally, patients with missing absolute lymphocyte count (Lym) values were also excluded from the analysis. The final study cohort comprised 317 patients diagnosed with DCM (*N* = 190), HCM (*N* = 114), or RCM (*N* = 13) (Table 1). Figure 1 illustrates the patient selection process and provides a detailed account of the ICD codes used to exclude cases with coexisting cardiomyopathies.

### 2.3. Data Extraction and Definitions

Data extraction from the MIMIC-IV database was conducted using PostgreSQL software (version 13.4; PostgreSQL Global Development Group, Berkeley, CA, USA), focusing on demographic characteristics, medication usage, prognostic indicators, comorbidities, disease severity scores, vital signs, and laboratory measurements. For laboratory data, only the first recorded values obtained after ICU admission were included in the analysis. The demographic characteristics encompassed age, sex, and body weight, while the laboratory parameters included Lym, absolute neutrophil count (Neu), absolute monocyte count (Mon), hematocrit, hemoglobin, absolute platelet count (PLT), red blood cell (RBC) count, white blood cell (WBC) count, and anion gap, among others. Disease severity was assessed using the sequential organ failure assessment (SOFA) score, acute physiology score III (APS III), simplified acute physiology score II (SAPS II), and Oxford acute severity of illness score (OASIS). Comorbidities considered in this study included hypertension, stroke, chronic kidney disease, cancer, type 2 diabetes, hyperlipidemia, heart failure, myocardial infarction, chronic obstructive pulmonary disease, mechanical ventilation, and sepsis. Vital sign data primarily consisted of non-invasive blood pressure (NIBP), respiratory rate, heart rate, and oxygen saturation (SpO₂). Medication usage included angiotensin-converting enzyme inhibitors (ACEI), angiotensin II receptor blockers (ARB), β-blockers, aldosterone antagonists, diuretics, inotropes, and anticoagulants. In this study, medication use—including β-blockers therapy—was operationally defined as any recorded administration during the ICU stay, regardless of dosage, specific compound, or treatment duration. Data on medication exposure prior to ICU admission were not available and, thus, not considered in the analysis. Consequently, β-blocker exposure herein refers solely to in-ICU administration, encompassing both newly initiated and ongoing therapy during ICU hospitalization. The ICD codes used for extracting each comorbidity are detailed in Supplementary Table S1, while the generic sequence numbers for medication extraction are provided in Supplementary Table S2. To address missing data, continuous variables with more than 15% missing rates were excluded from the analysis, while those with missing rates between 5% and 15% were imputed using multiple imputation techniques. For variables with less than 5% missing data, the mean of the available observations was used for imputation. A summary of variables with missing data included in this study is presented in Supplementary Table S3. The primary exposure variables were the SII, SIRI, AISI, and RDW. These indices were defined as follows: SII = (Neu× PLT)/Lym, SIRI = (Neu × Mon)/Lym, AISI = (Neu × PLT × Mon)/Lym, RDW = (Standard deviation of RBC volume/Mean corpuscular volume) × 100. The units for Lym, Neu, Mon, and PLT were all expressed as K/µL. Due to the large numerical values of the AISI and SII indices, their values were primarily analyzed as AISI/100 and SII/100, respectively, in subsequent analyses, unless otherwise specified. This transformation was performed to facilitate easier interpretation of regression coefficients and to improve the numerical stability of the statistical and machine learning models. Longitudinal RDW measurements were systematically collected over the course of ICU admission to ensure comprehensive assessment.

In this study, 365-day all-cause mortality was selected as the primary outcome rather than ICU or in-hospital mortality. This choice was based on the need to capture both the acute effects of ICU interventions and the longer-term consequences of underlying disease processes, including treatment adherence, recovery, and complications post discharge. Additionally, the number of events at 365 days (*n* = 52) was higher than those for ICU or in-hospital mortality, which helped to enhance statistical robustness and minimize bias due to low event rates in regression and machine learning models.

### 2.4. Statistical Analysis

Categorical variables were summarized as proportions. To describe continuous variables, we first assessed their distribution. Variables with approximately normal distributions were reported as means with standard deviations, whereas non-normally distributed variables were described using medians and interquartile ranges. Depending on data type and distribution, we applied suitable statistical tests for group comparisons, including Student’s *t*-test, Mann–Whitney U test, chi-square test, or Fisher’s exact test. We used LASSO regression to screen for variables most strongly associated with 365-day mortality in cardiomyopathy patients. The λ value was selected using the 1-standard error rule based on 10-fold cross-validation to obtain a simpler and more generalizable model. In parallel, the Boruta algorithm was employed to identify key predictors and assess the importance of inflammatory markers such as SII, SIRI, AISI, and RDW. Variables selected by LASSO were then entered into a multivariate Cox proportional hazards model. To assess model performance, we calculated receiver operating characteristic (ROC) curves. We further tested whether these core variables met the proportional hazards (PH) assumption using formal Cox PH tests. Time-dependent Cox regression was conducted for variables with suspected time-related effects. Additionally, we used logistic regression to analyze associations between inflammatory indices and mortality risk. Survival outcomes were evaluated using Kaplan–Meier curves and log-rank tests, while RCS was used to explore potential nonlinear relationships. Finally, subgroup analyses were visualized using forest plots to assess possible interaction effects across different patient characteristics. Univariate subgroup generalized linear model (GLM) analyses were conducted to evaluate the relationships between RDW, AISI, SIRI, and SII and 365-day all-cause mortality within the DCM, HCM, and RCM patient groups. Three models were constructed in this study: Model 1 included no covariate adjustments; Model 2 was adjusted for age, gender, weight, hypertension, cancer, type 2 diabetes, hyperlipidemia, heart failure, and myocardial infarction; and Model 3 was further adjusted for age, gender, weight, hypertension, cancer, type 2 diabetes, hyperlipidemia, heart failure, myocardial infarction, sepsis, ACEI, ARB, β-blockers, aldosterone antagonists, diuretics, inotropes, white blood cell count, anion gap, total calcium, chloride, glucose, potassium, sodium, INR, prothrombin time, partial thromboplastin time, blood urea nitrogen, and creatinine. All statistical analyses were conducted using R software (version 4.4.1; R Foundation for Statistical Computing, Vienna, Austria) and DecisionLinnc 1.0 software (China) [19]. A two-tailed *p*-value of less than 0.05 was considered statistically significant, and the results of multivariate logistic and Cox regression analyses were reported as odds ratios (OR) or hazard ratios (HR) with corresponding 95% confidence intervals (CI).

### 2.5. Machine Learning

The core variables selected through LASSO regression and the Boruta algorithm were incorporated into machine learning algorithms. The dataset was split into a training set and a test set at a 7:3 ratio, with the training set used for model development and the test set for evaluation.

We selected and compared nine commonly used machine learning methods, each representing a different algorithmic approach: (1) logistic regression (a linear baseline classifier); (2) random forest (RF), an ensemble of decision trees effective for nonlinear data; (3) extreme gradient boosting (XGBoost), which uses boosting and regularization to optimize model performance; (4) support vector machine (SVM), known for maximizing class separation margins; (5) gradient boosting tree, which builds trees sequentially to reduce bias; (6) LightGBM, a fast and memory-efficient gradient boosting implementation; (7) CatBoost, designed to handle categorical features efficiently; (8) multilayer perceptron (MLP), a simple deep learning model with one or more hidden layers; and (9) k-nearest neighbors (KNN), a non-parametric method based on distance to training examples. These methods were chosen to cover a wide spectrum of model interpretability, flexibility, and computational efficiency. During model development, the optimal hyperparameters were determined, and the detailed parameter settings for each model are provided in Supplementary Table S4.

To evaluate model performance, we calculated ROC curves and the corresponding area under the curve (AUC). Clinical utility was evaluated using decision curve analysis (DCA), while calibration curves were employed to evaluate the model’s accuracy in predicting absolute risk. To further interpret the most predictive model on the test set, SHAP values and feature selection tree models were employed, both of which provided insights into variable importance and ranking [20]. Additionally, cross-validation was performed to assess the robustness of the best-performing model, with evaluation metrics including ROC and DCA. The validation process involved five iterations, with the random seed set to 100. Furthermore, the bootstrap resampling method was applied with 10 iterations, a training sample proportion of 70%, and a random seed set to 500 to enhance the reproducibility of the results.

### 2.6. Analysis of Repeated-Measures Data

This study systematically collected longitudinal RDW measurements in patients with cardiomyopathy during ICU admission to ensure a comprehensive assessment. Grouped error trajectory trend curves were employed to explore the distribution and error trajectories of RDW across different time intervals within the first 30 days of ICU admission. Additionally, mean trajectory trend curves were plotted to visualize the temporal distribution patterns of RDW over the same period. To further investigate temporal changes, repeated-measures analysis of variance (ANOVA) was conducted to compare the mean differences across repeated observations within the same subjects, assess within-subject effects, and reduce variability caused by individual differences.

### 2.7. BMJM Development

The BMJM comprises a longitudinal submodel and a survival submodel [21,22,23,24]. In this study, a linear mixed-effects model was employed as the longitudinal submodel, while a Cox proportional hazards model was utilized as the survival submodel. The linear mixed-effects model preserves the normality assumptions of traditional linear models while relaxing the assumptions of independence and homoscedasticity, enabling the simultaneous analysis of both fixed and random effects, thereby enhancing the robustness and reliability of the results. The Cox proportional hazards model, widely recognized in survival analysis, serves as a semi-parametric model that does not impose assumptions on the distribution of event times, offering greater analytical flexibility and computational efficiency [25]. During the construction of the BMJM, observation time was treated as a time-dependent covariate, while RDW was designated as the dependent variable. A linear interaction between time-dependent covariates and β-blockers was incorporated, and the time-dependent covariate was modeled as a random effect in the longitudinal submodel. In the survival submodel, survival time and 365-day all-cause mortality served as dependent variables, with β-blockers serving as independent variables. The two submodels were linked through shared random effects to capture the correlation between longitudinal and survival outcomes. Within the Bayesian framework, non-informative priors were applied to streamline the model estimation process. The Markov chain Monte Carlo algorithm was employed for parameter estimation, ensuring computational robustness and efficiency. The model was set to run for 15,000 iterations, with a burn-in period of 1000 iterations, and three Markov chains were employed. The BMJM was visualized through time-varying effect curves, coefficient iteration trajectory plots, ROC curves, and calibration curves. The model’s predictive performance was evaluated by computing the AUC. Three patients were selected for regression prediction using the Bayesian multivariate joint model, and the predicted trajectories were visualized through trajectory plots.

## 3. Results

### 3.1. Baseline Characteristics

Based on the selection criteria, a total of 317 patients diagnosed with DCM (*N* = 190), HCM (*N* = 114), or RCM (*N* = 13) were included in this study (Figure 1). Table 1 summarizes the baseline characteristics of the survivor and non-survivor cohorts at 365 days. The survivor group comprised 265 patients, while the non-survivor group included 52 patients. Patients in the non-survivor group generally exhibited higher levels of SII, SIRI, AISI, and RDW, along with a higher proportion of females, anion gap, blood glucose, prothrombin time, partial thromboplastin time, international normalized ratio, blood urea nitrogen, and creatinine. Additionally, they had higher SOFA, APS III, SAPS II, and OASIS values, increased respiratory rate, and a higher proportion of inotrope usage. Conversely, survivors were more likely to have higher Lym and chloride levels, as well as a greater proportion of patients using ACEI and β-blockers. Furthermore, the prevalence of myocardial infarction and sepsis was lower among survivors, who also demonstrated lower in-hospital and ICU mortality rates. Supplementary Table S5 presents the clinical characteristics of DCM, HCM, and RCM. Among the three cardiomyopathy subtypes, RDW exhibited significant differences, with RCM patients demonstrating the highest RDW levels.

### 3.2. Primary Outcomes of Cox Regression Analyses

**Figure 2:**
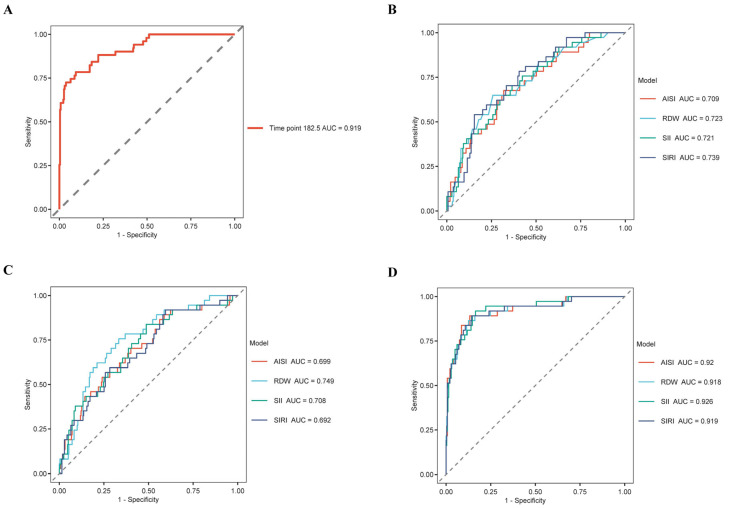
ROC curves of Cox regression analysis: (**A**) ROC curve of LASSO survival regression; (**B**) ROC curve of multivariate Cox regression analysis of Model 1; (**C**) ROC curve of multivariate Cox regression analysis of Model 2; (**D**) ROC curve of multivariate Cox regression analysis of Model 3. Abbreviations: ROC: receiver operating characteristic; AUC: area under the curve; RDW: red blood cell distribution width; SIRI: systemic inflammation response index; AISI: aggregate index of systemic inflammation; SII: systemic immune-inflammation index.

Next, LASSO regression was employed, as previously described, to identify the most representative risk factors associated with 365-day mortality in patients with cardiomyopathy (Supplementary Figures S2A,B, Figure 2A). LASSO regression identified 22 significant risk factors, including age, sex, RDW, and SII, based on the number of non-zero regression coefficients (Table 2). All 22 variables selected by LASSO were included in the final multivariate Cox regression model. As shown in Table 2, age, myocardial infarction, partial thromboplastin time, RDW, and SII levels were identified as independent risk factors for 365-day mortality in patients with cardiomyopathy, while sex, β-blockers, and aldosterone antagonists were identified as independent protective factors against 365-day mortality in these patients. The HR was 0.2 for β-blockers (95% CI: 0.1–0.39; *p* < 0.001), was 1.14 for RDW (95% CI: 1.01–1.29; *p* = 0.03), and 1.01 for SII (95% CI: 1.00–1.01; *p* = 0.03).

A multivariate Cox regression analysis was conducted, where Model 1 included the SII, SIRI, AISI, and RDW as individual univariate models, while Model 2 and Model 3 incorporated covariate adjustments as previously described. The predictive performance of these three models was evaluated using Cox multi-model ROC curves at day 30 (Figure 2B–D). The results demonstrated that in Model 3, which included a larger number of covariates, the AUC values were higher, indicating a more robust and stable model.

To assess the PH assumption, Cox PH tests were performed on 10 variables: age, sex, myocardial infarction, partial thromboplastin time, β-blockers, aldosterone antagonists, RDW, SII, SIRI, and AISI (Supplementary Figure S2), confirming that all variables met the PH assumption. Using these 10 variables as independent variables, a time-dependent Cox regression analysis was conducted, treating β-blockers, aldosterone antagonists, RDW, SII, SIRI, and AISI as time-dependent covariates. The results showed that the time-dependent HR for RDW was 1.20 (95% CI: 1.06–1.34; *p* = 0.003), for SIRI it was 1.01 (95% CI: 0.99–1.03; *p* = 0.24), for AISI it was 1.00 (95% CI: 0.99–1.01; *p* = 0.84), for SII it was 1.01 (95% CI: 0.99–1.02; *p* = 0.34), for β-blockers it was 0.23 (95% CI: 0.11–0.48; *p* = 0.0001), and for aldosterone antagonists it was 0.28 (95% CI: 0.07–1.07; *p* = 0.06).

### 3.3. Primary Outcomes of Logistic Regression Analyses

As shown in Table 3, the levels of RDW, SII, SIRI, and AISI were all significantly associated with 365-day mortality in patients with cardiomyopathy. Model 1 did not include any covariate adjustments, while Model 2 and Model 3 incorporated adjustments as previously described. The analysis demonstrated that in Model 3, RDW levels remained positively associated with 365-day mortality in cardiomyopathy patients (OR: 1.31, 95% CI: 1.10–1.60, *p* = 0.004). Subsequently, RDW, SII, SIRI, and AISI levels were categorized into three quantiles, with Q1 serving as the reference group. The analysis revealed that across all models, the 365-day mortality risk in cardiomyopathy patients was significantly higher in the highest quantile (Q3) compared to Q2 for RDW, SII, SIRI, and AISI levels. After adjusting for covariates and performing quantile stratification, the overall *p* for trend for all four biomarkers remained statistically significant, indicating a robust association.

### 3.4. KM Survival Analyses

**Figure 3:**
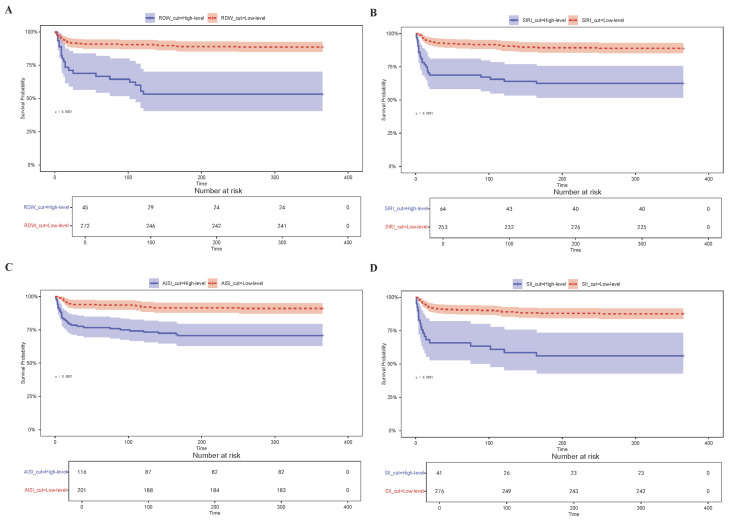
KM survival curves of RDW, SIRI, AISI, and SII levels for 365-day mortality in cardiomyopathy patients admitted to the ICU. (**A**) KM survival curves of RDW levels; (**B**) KM survival curves of SIRI levels; (**C**) KM survival curves of AISI levels; (**D**) KM survival curves of SII levels; RDW: red blood cell distribution width; SIRI: systemic inflammation response index; AISI: aggregate index of systemic inflammation; SII: systemic immune-inflammation index; ICU: intensive care unit.

Subsequently, KM survival analysis was performed based on RDW, SII, SIRI, and AISI levels to evaluate 365-day mortality in patients with cardiomyopathy (Figure 3A–D). Through the optimal cut-off point analysis, the optimal thresholds for predicting 365-day mortality were identified as 18% for RDW, 13.15 for SII, 13.06 for SIRI, and 44.74 for AISI. The proportion of patients in the high-level group was lower for RDW, SII, and SIRI, whereas the proportion was higher for AISI. Moreover, compared with patients with lower RDW, SII, SIRI, and AISI levels, those with elevated levels exhibited significantly lower survival rates (*p* < 0.0001) (Figure 3A–D).

### 3.5. Nonlinear Analyses

**Figure 4:**
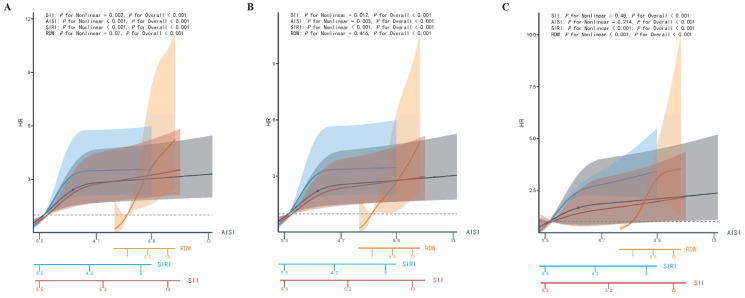
RCS analysis of RDW, SIRI, AISI, and SII levels for 365-day mortality in cardiomyopathy patients admitted to the ICU. (**A**) RCS analysis of Model 1; (**B**) RCS analysis of Model 2; (**C**) RCS analysis of Model 3. Abbreviations: RCS: Restricted cubic spline; RDW: red blood cell distribution width; SIRI: systemic inflammation response index; AISI: aggregate index of systemic inflammation; SII: systemic immune-inflammation index; ICU: intensive care unit; HR: hazard ratios.

As previously described, RCS analysis was conducted with Model 1, treating SII, SIRI, AISI, and RDW as independent univariate models, while Model 2 and Model 3 incorporated covariate adjustments, as outlined earlier. To facilitate direct comparison and ensure that the curves for all four biomarkers were plotted on a common *x*-axis scale, RDW, SIRI, AISI, and SII were rescaled by dividing by 2, 5, 1000, and 1000, respectively. The RCS analysis revealed that, in Model 1 and Model 2, there were strong nonlinear relationships between SII, SIRI, and AISI and 365-day mortality in cardiomyopathy patients, whereas RDW exhibited a strong linear relationship with 365-day mortality (Figure 4A,B). In Model 3, a strong nonlinear relationship was observed among SIRI, RDW, and 365-day mortality, while SII and AISI demonstrated a strong linear relationship (Figure 4C). Across all three RCS models, RDW demonstrated a markedly higher HR for 365-day mortality in cardiomyopathy patients compared to SII, SIRI, and AISI. Supplementary Figure S3 presents the Model 1 RCS curve analyses for SII, SIRI, AISI, and RDW in DCM and HCM, highlighting distinct curve patterns between the two conditions. Specifically, the HR values for SII, SIRI, and AISI were generally higher in HCM, whereas RDW exhibited a higher HR in DCM.

### 3.6. Subgroup Interaction Regression Analysis

Based on the results of LASSO regression, a subgroup interaction analysis was conducted to examine the relationships between SII, SIRI, AISI, and RDW, and 365-day all-cause mortality in patients with cardiomyopathy, while also assessing whether these relationships were modified by age, sex, myocardial infarction, partial thromboplastin time, β-blockers, and aldosterone antagonists. Although the OR varied across different patient subgroups, the overall associations between SII, SIRI, AISI, and RDW, and 365-day all-cause mortality remained positively correlated. The interaction analysis further indicated that the use of β-blockers and aldosterone antagonists, as well as patient characteristics such as age, sex, myocardial infarction, and partial thromboplastin time, did not significantly alter these associations (Supplementary Figure S4A–D). Univariate subgroup GLM analyses revealed that the associations between AISI, SIRI, and SII and 365-day mortality were relatively consistent across the DCM, HCM, and RCM subgroups as well as the overall cohort, with DCM patients contributing most significantly to the observed mortality risk. In contrast, RDW showed a strong and significant association with 365-day mortality in the DCM subgroup, whereas no significant associations were observed in the HCM and RCM subgroups (Supplementary Figure S4E).

### 3.7. Boruta Algorithm

**Figure 5:**
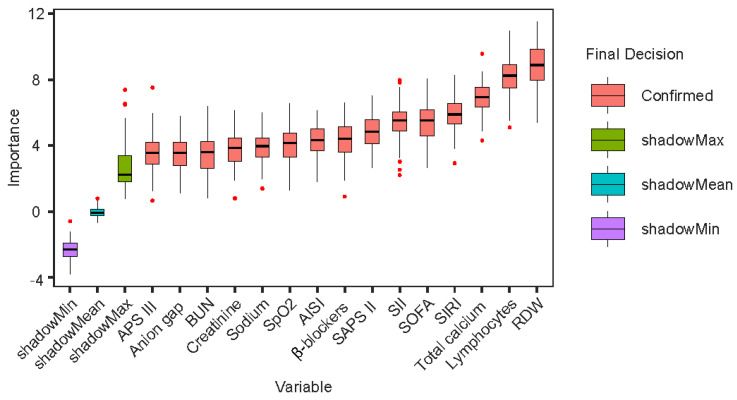
The results of the Boruta algorithm. Abbreviations: RDW: red blood cell distribution width; SIRI: systemic inflammation response index; AISI: aggregate index of systemic inflammation; SII: systemic immune-inflammation index; SOFA: sequential organ failure assessment; APS III: acute physiology scores III; SAPS II: simplified acute physiology score II; SpO₂: oxygen saturation; BUN: blood urea nitrogen.

In this study, the Boruta algorithm was employed to identify the most important feature variables within the dataset. This algorithm determines feature importance by comparing the Z-score of each actual feature with that of corresponding “shadow features”. Specifically, all real features are duplicated and randomly shuffled, after which an RF model is used to compute the Z-score for each feature. Additionally, the Z-scores of the “shadow features” are generated by randomly permuting the original features [26]. Figure 5 presents the feature selection results based on the Boruta algorithm, indicating that RDW had the highest importance value among the core variables, followed by SIRI and SII, with AISI ranking last. Among the non-core variables, Lym, total calcium, and several disease severity scores exhibited relatively high importance values.

### 3.8. Establishment and Validation of the Prediction Models

**Figure 6:**
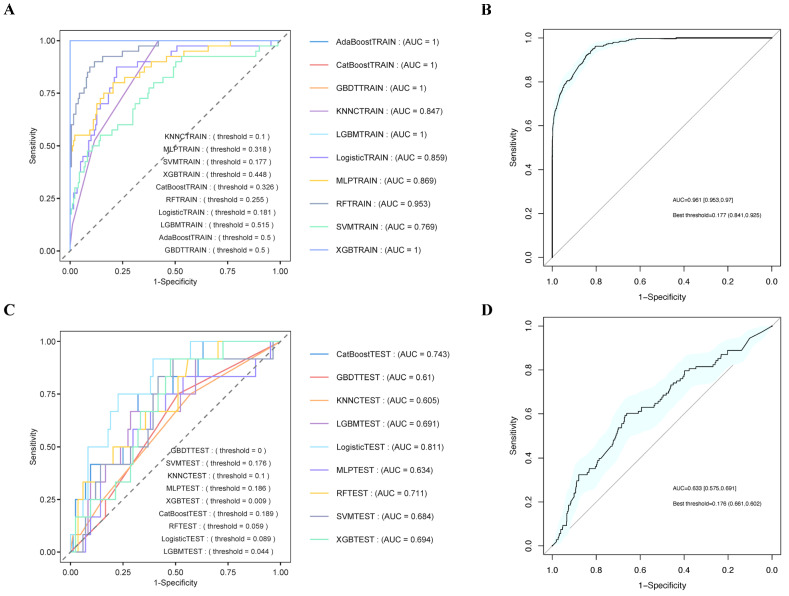
The ROC curves for the machine learning. (**A**) ROC curves of the training sets; (**B**) ROC curve of the training sets with a 95% CI; (**C**) ROC curves of the testing sets; (**D**) ROC curve of the testing sets with a 95% CI. Abbreviations: ROC: receiver operating characteristic; AUC: area under the curve; RF: random forest; XGB: extreme gradient boosting survival learner; SVM: support vector machine; MLP: multilayer perceptron; KNN: k-nearest neighbors; GBDT: gradient boosting tree; LGBM: LightGBM; CI: confidence interval.

**Figure 7:**
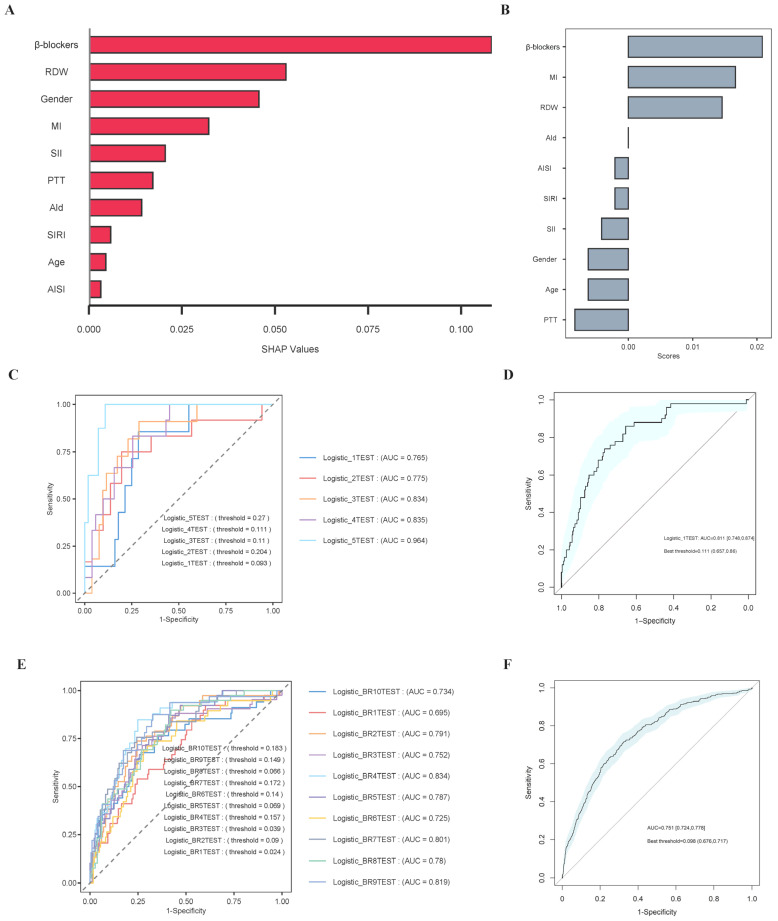
The results of SHAP analysis, feature selection tree models, and cross-validation. (**A**) The results of SHAP analysis; (**B**) the results of feature selection tree models; (**C**) ROC curves of cross-validation after 5 cycles; (**D**) ROC curves of cross-validation with a 95% CI; (**E**) ROC curves of bootstrap analysis after 10 cycles; (**F**) ROC curves of bootstrap analysis with a 95% CI. Abbreviations: RDW: red blood cell distribution width; MI: myocardial infarction; SII: systemic immune-inflammation index; PTT: partial thromboplastin time; Ald: Aldosterone antagonists; SIRI: systemic inflammation response index; AISI: aggregate index of systemic inflammation; ROC: receiver operating characteristic; AUC: area under the curve; CI: confidence interval.

The core variables (age, sex, myocardial infarction, partial thromboplastin time, β-blockers, aldosterone antagonists, RDW, SII, AISI, and SIRI levels) selected through LASSO regression and the Boruta algorithm were incorporated into machine learning algorithms. We applied machine learning techniques to predict 365-day mortality in cardiomyopathy patients. Figure 6A,B present the ROC curves for both the training and test sets, with model performance evaluated based on AUC values. Supplementary Figure S5A–D present the DCA and calibration curves for the training and test sets, indicating that all models exhibited good clinical utility and predictive accuracy. As shown in Figure 6C,D, the AUC and 95% CI in the test set (AUC: 0.633, 95% CI: 0.575–0.691) were lower than those in the training set (AUC: 0.961, 95% CI: 0.953–0.97). Among the models tested in the test set, logistic classification achieved the highest AUC (0.811), making it the best-performing model based on AUC results. SHAP analysis applied to the logistic classification model highlighted RDW and β-blockers as the most crucial variables (Figure 7A), underscoring their substantial contribution to the model’s predictions. Furthermore, feature selection using tree model analysis in the logistic classification model also identified RDW and β-blockers as the most influential variables (Figure 7B), further emphasizing their key role in predictive outcomes.

To further assess the logistic classification model’s performance, cross-validation was conducted. Supplementary Figure S5E,F presents the DCA and calibration curves from the cross-validation analysis. Figure 7C shows that the AUC values for all models exceeded 0.7, while Figure 7D indicates that the 95% CI for AUC ranged from 0.748 to 0.874, confirming that these models outperformed random guessing. Notably, the Logistic_5TEST and Logistic_4TEST models demonstrated the best performance, achieving relatively high AUC values. Supplementary Figure S5G,H presents the DCA and calibration curves from the bootstrap analysis. Figure 7E shows that the AUC values for all models were nearly all above 0.7, while Figure 7F demonstrates that the 95% confidence intervals (CIs) for the AUC ranged from 0.724 to 0.778. Notably, the Logistic_BR4TEST and Logistic_BR9TEST models exhibited the best performance, achieving relatively high AUC values.

### 3.9. The Results of Analysis of Repeated-Measures Data

Based on the time-dependent Cox regression analysis, RDW and β-blockers demonstrated statistical significance. Additionally, results from SHAP analysis and feature selection using tree model analysis in machine learning consistently identified RDW and β-blockers as the most influential variables. Given the characteristics of the MIMIC database, which contains extensive repeated RDW measurements for cardiomyopathy patients during ICU admission, this study systematically collected longitudinal RDW data throughout the ICU stay to ensure a comprehensive evaluation.

**Figure 8:**
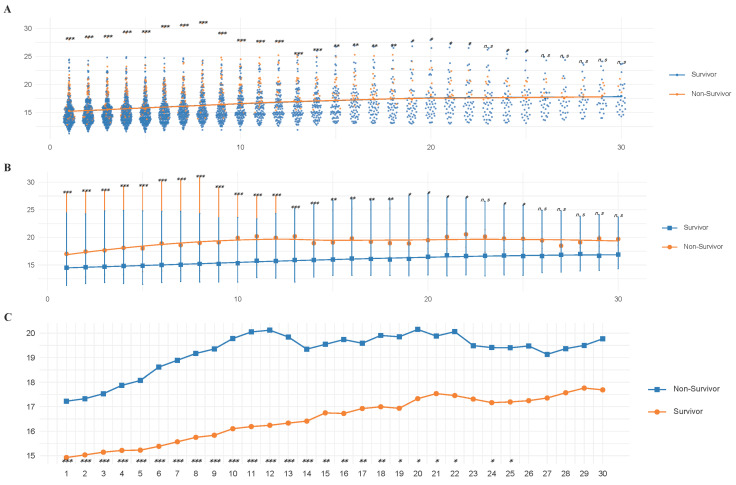
The results of trajectory trend curves. (**A**) Group distribution trajectory trend curve; (**B**) Group error trajectory trend curve; (**C**) Group mean trajectory trend curve. * represent *p* < 0.05; ** represent *p* < 0.01; *** represent *p* < 0.001; n.s represent not significant.

**Figure 9:**
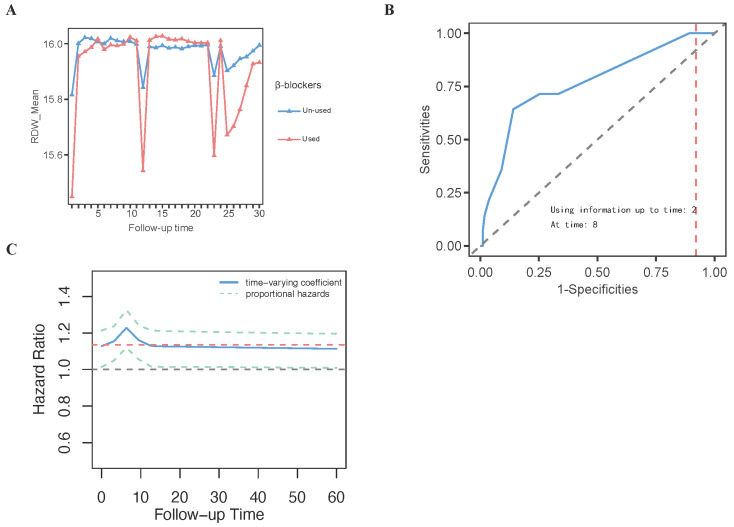
The results of repeated measures ANOVA and the BMJM. (**A**) The results of repeated measures ANOVA; (**B**) The ROC curves of the BMJM; The red dashed line denotes the cut-off threshold on the ROC curve for predicting outcomes at time = 8 using data up to time = 2; (**C**) The time-varying effect curves of the BMJM; The red dashed line represents the estimated constant hazard ratio under the proportional hazards assumption. Abbreviations: RDW: red blood cell distribution width; BMJM: Bayesian multivariate joint model; ROC: receiver operating characteristic; ANOVA: analysis of variance.

Grouped error trajectory trend curves and mean trajectory trend curves depicting RDW distribution patterns and error trajectories over different time intervals within the first 30 days of ICU admission revealed that RDW levels exhibited a more pronounced upward trend in the non-survivor group compared to the survivor group (Figure 8A–C). Moreover, RDW values progressively increased with prolonged hospitalization (Figure 8A–C). To further investigate the temporal variations in RDW, repeated-measures ANOVA was conducted, with RDW as the dependent variable, continuous monitoring time as the within-subject variable, and β-blockers as the between-subject variable. The analysis revealed a statistically significant interaction between RDW trends and β-blocker therapy (*p* < 0.0001, *F* = 6.65), indicating that the trajectory of RDW over time differed significantly between patients who received β-blockers and those who did not. As shown in Figure 9A, patients receiving β-blockers exhibited a notable and consistent decrease in RDW levels during ICU stay, suggesting a potential beneficial effect of β-blocker therapy on inflammatory or hematologic stability. These findings support the use of RDW as a dynamic biomarker for monitoring therapeutic response to β-blockers in critically ill patients with cardiomyopathy.

### 3.10. The Results of BMJM Development

**Figure 10:**
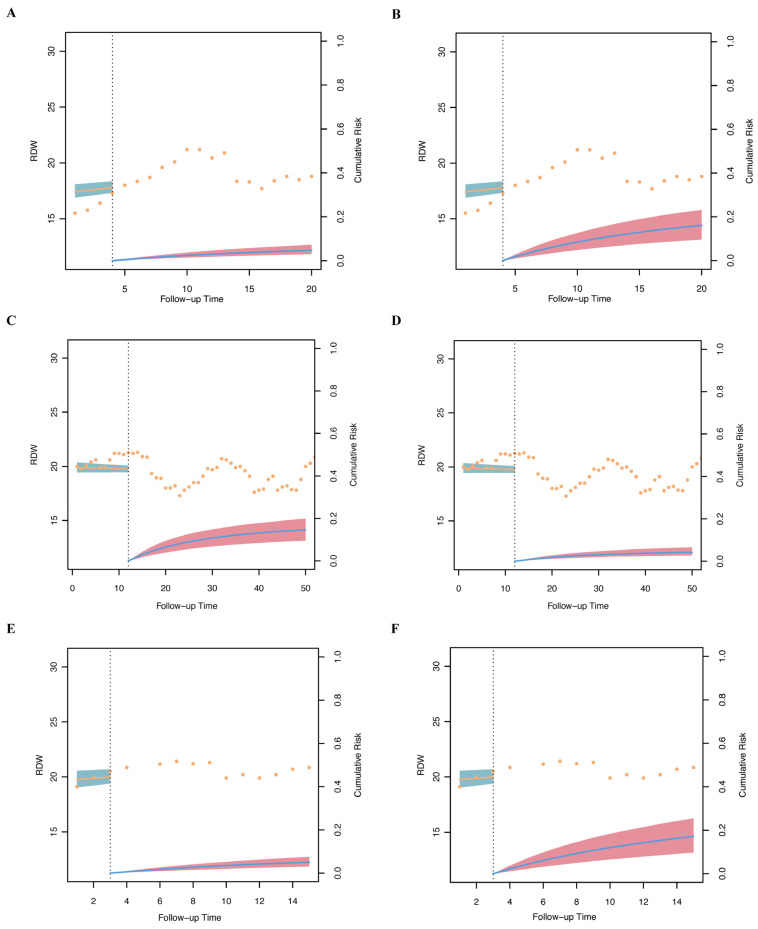
Regression model prediction results for three patients: (**A**) Prediction of the outcome of patient 1’s original medication; (**B**) Prediction of the outcome of patient 1 changing medication; (**C**) Prediction of the outcome of patient 2’s original medication; (**D**) Prediction of the outcome of patient 2 changing medication; (**E**) Prediction of the outcome of patient 3’s original medication; (**F**) Prediction of the outcome of patient 3 changing medication. Orange dots indicate the observed RDW values. The light blue band represents the fitted RDW trajectory with its 95% confidence interval. The blue curve in the red shaded area shows the predicted cumulative mortality risk, and the red band indicates its 95% confidence interval. The vertical dotted line marks the intervention time point (e.g., initiation or discontinuation of β-blocker therapy). Abbreviations: RDW: red blood cell distribution width.

Following the methodology described above, we developed the BMJM, which achieved an AUC of 0.80 and a Youden index of 0.96, indicating high predictive performance (Figure 9B). The time-varying effect curves demonstrated that the time-varying coefficient of RDW and proportional hazards peaked at approximately day 7 before stabilizing (Figure 9C). Both the calibration curve (Supplementary Figure S5I) and the coefficient iteration trajectory plot (Supplementary Figure S6) confirmed the robustness of the BMJM. Subsequently, three patients were selected for regression prediction using the BMJM. Patient 1 was a 62-year-old female with obstructive HCM, who was receiving β-blocker therapy as part of standard treatment but experienced ICU mortality at day 30. Using the BMJM, an intervention was simulated on day 4 of ICU admission, where β-blocker therapy was discontinued. Under this modification, the patient’s predicted mortality risk by day 20 increased significantly, rising from 4.73% with β-blocker use to 16.1% without β-blockers (Figure 10A,B). Patient 2 was a 54-year-old female with DCM, who did not receive β-blockers during her standard treatment and experienced ICU mortality at day 56. Using BMJM, an intervention was simulated on day 12 of ICU admission, where β-blockers were introduced. Under this modification, the patient’s predicted mortality risk by day 50 decreased significantly, dropping from 14.65% without β-blockers to 4.26% with β-blockers (Figure 10C,D). Patient 3 was a 45-year-old female with RCM, who was receiving β-blocker therapy but experienced ICU mortality at day 19. Using the BMJM, an intervention was simulated on day 3 of ICU admission, where β-blocker therapy was discontinued. Under this modification, the patient’s predicted mortality risk by day 15 increased significantly, rising from 6.08% with β-blockers to 17.16% without β-blockers (Figure 10E,F). These findings demonstrate that the BMJM, by monitoring dynamic changes in RDW, can predict future mortality risk in cardiomyopathy patients based on whether β-blockers are administered.

## 4. Discussion

This study revealed that RDW was a robust independent predictor of 365-day all-cause mortality in ICU-admitted patients with cardiomyopathy, while β-blocker therapy was identified as a strong independent protective factor. These associations remained significant in both standard and time-dependent Cox regression models, confirming their temporal relevance. Machine learning models further validated these findings: logistic classification achieved the best predictive performance, and both SHAP and tree-based feature selection consistently identified RDW and β-blockers as the most influential predictors. Comparative analyses using RCS demonstrated that RDW had a stronger and more linear association with 365-day mortality than other inflammatory indices (SII, SIRI, AISI). Longitudinal trend analyses showed a progressive increase in RDW among non-survivors. Repeated measures ANOVA confirmed a significant interaction between RDW dynamics and β-blocker therapy, with RDW levels significantly reduced in patients receiving β-blockers. The BMJM further demonstrated that incorporating RDW dynamics and β-blocker use enables accurate prediction of mortality risk. Simulated discontinuation of β-blockers markedly increased mortality risk, while simulated initiation reduced it, underscoring the clinical utility of RDW as a dynamic biomarker for treatment monitoring and individualized risk stratification.

Previous studies have collectively highlighted the significant prognostic value of RDW in patients with DCM and HCM. RDW has demonstrated incremental predictive value for adverse clinical events beyond late gadolinium enhancement on cardiac magnetic resonance imaging in patients with non-ischemic DCM [27]. An independent association between RDW and impaired coronary flow reserve (CFR) has been observed in patients with idiopathic DCM, suggesting RDW as a reliable predictor of low CFR [28]. Additionally, RDW’s standard deviation was independently associated with poor outcomes in DCM patients with pre-diabetes and diabetes, but not in those with normoglycemia, underscoring its potential for personalized risk stratification in DCM subpopulations [29]. An RDW ≥ 15% has been identified as a marker for long-term mortality or heart transplantation in DCM patients with a left ventricular ejection fraction <30%, highlighting RDW’s prognostic role in advanced heart failure [11]. Furthermore, elevated RDW predicted mortality in DCM-related heart failure patients, reinforcing RDW’s importance as a prognostic biomarker in DCM and its potential integration into multi-marker risk models [12]. Elevated RDW has been associated with all-cause mortality and HCM-related death, with a saturation effect observed at an RDW level of approximately 14% [13]. RDW has also been identified as an independent predictor of heart failure hospitalization in HCM patients, with a cut-off value of 14% demonstrating high sensitivity and specificity [30]. Additionally, elevated RDW, together with maximum left ventricular wall thickness, independently predicted major adverse cardiac events and mortality in HCM patients [31]. In patients with hypertrophic obstructive cardiomyopathy (HOCM) undergoing septal myectomy, higher RDW levels were associated with increased all-cause and cardiovascular mortality, highlighting RDW as an additive biomarker for risk stratification in this population [32]. Previous studies have primarily focused on the prognostic role of RDW in HF associated with DCM or HCM or on the correlation between RDW and other clinical parameters in DCM and HCM. In contrast, the present study differs from prior research in several key respects. First, our study incorporated both cross-sectional RDW data and longitudinal repeated RDW measurements, enabling a comprehensive investigation of the risk association between RDW and mortality in DCM, HCM, and RCM. The findings demonstrated that RDW served as a strong prognostic indicator of mortality across all three cardiomyopathy subtypes. Additionally, our study examined the predictive relationship between RDW and mortality based on β-blocker usage, indicating that monitoring dynamic changes in RDW can help predict future mortality risk in cardiomyopathy patients, depending on β-blocker administration. Furthermore, our study compared the prognostic value of RDW with that of other inflammatory markers, including the AISI, SII, and SIRI, in predicting cardiomyopathy-related mortality. Through LASSO regression, the Boruta algorithm, machine learning techniques, SHAP analysis, and feature selection using tree-based models, RDW was identified as a stronger predictor of cardiomyopathy-related mortality compared to the AISI, SII, and SIRI.

The elevation of RDW in cardiomyopathy may reflect underlying pathophysiological changes, including chronic low-grade inflammation, impaired erythropoiesis, and iron metabolism disorders. Inflammatory cytokines such as interleukin-6 and tumor necrosis factor-α can interfere with erythroid progenitor cell maturation and promote ineffective erythropoiesis, resulting in increased red blood cell size variability [33,34]. Additionally, inflammation can affect iron availability through the hepcidin pathway, further exacerbating anisocytosis [35,36]. These mechanisms are consistent with observations in heart failure and other cardiovascular diseases [37,38], suggesting that RDW may act as an integrative marker of systemic stress, hematopoietic imbalance, and disease severity in cardiomyopathy patients.

Previous studies collectively demonstrated that the AISI, SII, and SIRI were significant inflammatory biomarkers associated with the prognosis of various cardiovascular diseases. Elevated AISI levels have been associated with increased cardiovascular mortality in patients with hypertension [39]. Higher AISI levels also predicted major adverse cardiovascular and cerebrovascular events and mortality in patients with acute myocardial infarction [40]. A positive correlation has been observed between AISI, SII, and SIRI and the occurrence of HF [14], with higher SIRI levels independently associated with an increased risk of HF [41]. Elevated SII and SIRI levels have been linked to all-cause and cardiovascular mortality in a 20-year cohort study [42]. Additionally, the SIRI and SII were associated with increased in-hospital and long-term mortality in patients with chronic HF, with the SIRI demonstrating superior prognostic value compared to C-reactive protein [15]. The SII has also been identified as an independent predictor of all-cause mortality in patients with HCM [16]. Lastly, high SII values were significantly associated with long-term mortality in patients with non-ischemic cardiomyopathy [43]. These findings suggested that the AISI, SII, and SIRI can serve as accessible and reliable biomarkers for risk stratification and prognostication in cardiovascular diseases. Previous studies have primarily explored the relationships between the AISI, SII, and SIRI and cardiovascular diseases, with limited research on their associations with cardiomyopathies. This study focused on investigating the relationships between the AISI, SII, and SIRI and 365-day all-cause mortality in patients with DCM, HCM, and RCM, demonstrating that the AISI, SII, and SIRI served as reliable prognostic indicators of 365-day all-cause mortality in patients with these three types of cardiomyopathies.

Traditional perspectives have suggested that β-blockers may exacerbate HF; however, clinical studies have demonstrated their ability to improve cardiac function, reduce the need for transplantation, and even enhance survival rates. As early as 1975, Waagstein et al. first reported that β-blockers could be used to treat heart failure caused by idiopathic DCM and improve cardiac function. Since then, numerous studies have confirmed the benefits of β-blockers in HF of various etiologies [44]. Subsequent research has shown that β-blockers reduce sympathetic nervous system activity, decrease myocardial oxygen consumption, improve the left ventricular ejection fraction, and enhance cardiac output. They can also lower the heart rate, alleviate cardiac workload, and mitigate left ventricular remodeling. Long-term use of β-blockers has been shown to improve left ventricular systolic function and slow disease progression [45,46]. Currently, ACEI and β-blockers are considered cornerstones of pharmacological therapy for chronic heart failure [47]. In summary, the application of β-blockers in cardiomyopathy has been widely recognized. However, future research should further explore individualized treatment strategies based on specific clinical conditions to optimize the clinical efficacy and safety of β-blocker therapy. Therefore, in the context of personalized treatment, this study found that administering β-blockers to cardiomyopathy patients with elevated RDW can effectively reduce mortality, highlighting the potential therapeutic benefits of β-blocker therapy in this patient population.

A distinctive feature of this study was the adoption of a contemporary BMJM method. The BMJM is a statistical method that integrates longitudinal data, such as repeated biomarker measurements or clinical parameters, with time-to-event data in order to provide dynamic and personalized risk predictions; it has been applied in various clinical domains, including predicting mortality and transplant needs in liver disease [48], allograft survival in kidney transplant recipients [49], and ventilatory-inefficiency-related mortality in acute respiratory distress syndrome [50]. Additionally, the BMJM has been utilized to assess the impact of gut microbiota diversity on ICU mortality [51], evaluate the association between urea levels and protein intake in critical illness [52], and examine how mechanical ventilation parameters influence survival in acute respiratory failure [53]. Joint models have also been employed to investigate the relationship between the neurological pupil index and intracranial pressure [54] and to highlight the prognostic significance of the urea-to-creatinine ratio in prolonged critical illness [55]. This modeling approach enables continuous, real-time updates to risk predictions, making it a valuable tool for precision medicine and dynamic clinical decision-making across diverse medical specialties. This study also utilized the BMJM method to integrate RDW dynamics and β-blocker therapy, monitored RDW fluctuations, and analyzed β-blocker usage to predict future mortality risk in cardiomyopathy patients, providing a valuable tool for personalized clinical decision making.

### Limitations

This study was the first to utilize longitudinal dynamic ICU data on RDW to explore its role in monitoring β-blocker therapy and its association with 365-day mortality in cardiomyopathy. The study population consisted of a large cohort of ICU-admitted patients with DCM, HCM, and RCM. The primary strength of this study lies in its use of data from the publicly available MIMIC-IV database, enabling a comprehensive analysis; however, several limitations should be acknowledged. First, as this study was based on single-center data from Beth Israel Deaconess Medical Center in the United States, the external validity of our findings is limited. The specific ICU protocols, patient management practices, and regional prescribing patterns—particularly regarding β-blocker use—may not reflect those in other institutions or regions globally. Selection bias may also arise due to the retrospective nature of the study and the inclusion criteria derived from the MIMIC-IV database. Therefore, our results should be interpreted with caution, and future prospective, multicenter studies across diverse geographic and healthcare settings are needed to validate our findings and enhance generalizability. Second, due to the study design, we are unable to establish a causal relationship between the inflammatory markers RDW, AISI, SII, and SIRI, and 365-day mortality in cardiomyopathy patients, highlighting the need for further research in this area. Third, our study focused on critically ill DCM, HCM, and RCM patients admitted to the ICU, which may limit the generalizability of our findings to broader cardiomyopathy populations, particularly those receiving care in outpatient clinics or rapid diagnostic centers with milder disease presentations. Future research should include both outpatient and inpatient cohorts to compare outcomes and provide a better understanding of mortality risk across different care settings. Fourth, while we accounted for multiple potential confounders, the possibility of residual confounding cannot be entirely ruled out. However, we employed a BMJM method, which robustly integrates repeated measurements, maximizing the use of longitudinal data. This approach establishes conditional independence between longitudinal measurements and time-to-event outcomes using subject-specific random-effects structures, thereby addressing informative censoring due to mortality and adjusting for time-varying disease severity as a potential confounder. Fifth, although we adopted multiple widely accepted machine learning algorithms to predict mortality and improve model interpretability, we did not include modern deep learning architectures such as convolutional neural networks (CNNs) or transformer-based models. This decision was made considering the limited sample size, particularly in the RCM subgroup, and the tabular nature of the data, which may not fully benefit from deep learning’s capacity for high-dimensional feature extraction. Moreover, deep learning models tend to require larger datasets and carry a higher risk of overfitting in smaller clinical cohorts. Nonetheless, future work could explore the integration of attention mechanisms or hybrid architectures to further enhance prediction accuracy and capture complex temporal dependencies. Finally, it is important to note that the RCM subgroup in our cohort was relatively small, which may limit the robustness of subgroup-specific conclusions. However, sensitivity analyses excluding RCM cases showed consistent results, supporting the overall validity of our findings. Looking forward, future studies should include larger and more diverse cohorts, particularly with expanded RCM representation and multicenter data sources, to enhance generalizability. Additional investigations are warranted to determine the optimal frequency and timing of RDW monitoring in ICU settings and to evaluate how β-blocker dosing influences RDW dynamics and patient outcomes.

## 5. Conclusions

Our findings underscore the importance of dynamic RDW monitoring in ICU-admitted cardiomyopathy patients and its integration with β-blocker therapy into personalized treatment strategies. The use of the BMJM to analyze longitudinal biomarker data, incorporating RDW dynamics and β-blocker administration, offers a promising approach for enhancing risk stratification and improving clinical outcomes in cardiomyopathy patients requiring intensive care.

## Figures and Tables

**Table 1:** Characteristics of study participants.

Variable	Total (*n* = 317)	Survivor (*n* = 265)	Non-Survivor (*n* = 52)	*p*-Value
Age, years	65.00 (56.00–75.00)	65.00 (54.00–75.00)	68.50 (61.00–77.00)	0.069
Weight, kg	85.10 (71.40–100.20)	85.80 (71.55–101.40)	80.65 (69.55–94.38)	0.197
Gender				**0.02**
Female	97.00 (30.60%)	74.00 (27.92%)	23.00 (44.23%)	
Male	220.00 (69.40%)	191.00 (72.08%)	29.00 (55.77%)	
Disease classification, *n* (%)				0.601
DCM	190.00 (59.94%)	157.00 (59.25%)	33.00 (63.46%)	
HCM	114.00 (35.96%)	98.00 (36.98%)	16.00 (30.77%)	
RCM	13.00 (4.10%)	10.00 (3.77%)	3.00 (5.77%)	
Hypertension, *n* (%)				0.758
No	251.00 (79.18%)	209.00 (78.87%)	42.00 (80.77%)	
Yes	66.00 (20.82%)	56.00 (21.13%)	10.00 (19.23%)	
Stroke, *n* (%)				0.166
No	289.00 (91.17%)	239.00 (90.19%)	50.00 (96.15%)	
Yes	28.00 (8.83%)	26.00 (9.81%)	2.00 (3.85%)	
Chronic kidney disease, *n* (%)				0.172
No	226.00 (71.29%)	193.00 (72.83%)	33.00 (63.46%)	
Yes	91.00 (28.71%)	72.00 (27.17%)	19.00 (36.54%)	
Cancer, *n* (%)				0.431
No	284.00 (89.59%)	239.00 (90.19%)	45.00 (86.54%)	
Yes	33.00 (10.41%)	26.00 (9.81%)	7.00 (13.46%)	
Type 2 diabetes, *n* (%)				0.524
No	225.00 (70.98%)	190.00 (71.70%)	35.00 (67.31%)	
Yes	92.00 (29.02%)	75.00 (28.30%)	17.00 (32.69%)	
Hyperlipidemia, *n* (%)				0.232
No	152.00 (47.95%)	131.00 (49.43%)	21.00 (40.38%)	
Yes	165.00 (52.05%)	134.00 (50.57%)	31.00 (59.62%)	
Heart failure, *n* (%)				0.823
No	77.00 (24.29%)	65.00 (24.53%)	12.00 (23.08%)	
Yes	240.00 (75.71%)	200.00 (75.47%)	40.00 (76.92%)	
Myocardial infarction, *n* (%)				**0.001**
No	276.00 (87.07%)	238.00 (89.81%)	38.00 (73.08%)	
Yes	41.00 (12.93%)	27.00 (10.19%)	14.00 (26.92%)	
Chronic obstructive pulmonary disease, *n* (%)				0.482
No	272.00 (85.80%)	229.00 (86.42%)	43.00 (82.69%)	
Yes	45.00 (14.20%)	36.00 (13.58%)	9.00 (17.31%)	
Ventilation, *n* (%)				0.291
No	66.00 (20.82%)	58.00 (21.89%)	8.00 (15.38%)	
Yes	251.00 (79.18%)	207.00 (78.11%)	44.00 (84.62%)	
Sepsis, *n* (%)				**<0.001**
No	180.00 (56.78%)	168.00 (63.40%)	12.00 (23.08%)	
Yes	137.00 (43.22%)	97.00 (36.60%)	40.00 (76.92%)	
Hospital mortality, *n* (%)				**<0.001**
No	310.00 (97.79%)	265.00 (100.00%)	45.00 (86.54%)	
Yes	7.00 (2.21%)	0.00 (0.00%)	7.00 (13.46%)	
ICU mortality, *n* (%)				**<0.001**
No	271.00 (85.49%)	262.00 (98.87%)	9.00 (17.31%)	
Yes	46.00 (14.51%)	3.00 (1.13%)	43.00 (82.69%)	
LOS of hospital, day	10.17 (6.10–16.72)	9.99 (6.05–15.94)	12.11 (7.00–18.74)	0.369
LOS of ICU, day	2.68 (1.30–5.29)	2.22 (1.23–4.43)	7.15 (3.10–11.43)	**<0.001**
SOFA	5.00 (3.00–7.00)	5.00 (3.00–7.00)	7.00 (5.00–11.00)	**<0.001**
APS III	44.00 (33.00–56.00)	42.00 (32.00–53.00)	56.00 (48.50–74.00)	**<0.001**
SAPS II	36.00 (29.00–44.00)	35.00 (27.00–42.00)	45.00 (34.00–53.50)	**<0.001**
OASIS	30.00 (25.00–35.00)	29.00 (25.00–34.00)	34.00 (29.00–39.50)	**<0.001**
Lymphocytes, K/µL	1.32 (0.80–1.96)	1.43 (0.89–2.18)	0.76 (0.46–1.25)	**<0.001**
Neutrophils, K/µL	9.18 (5.50–12.65)	9.00 (5.39–11.87)	9.63 (5.81–14.09)	0.349
Monocytes, K/µL	0.76 (0.49–1.10)	0.75 (0.48–1.02)	0.86 (0.59–1.25)	0.093
Hematocrit, %	32.10 (27.60–37.50)	31.90 (27.60–37.50)	32.30 (26.95–38.25)	>0.999
Hemoglobin, g/dL	10.50 (8.80–12.00)	10.50 (8.90–12.10)	10.00 (8.20–12.00)	0.473
Platelet, K/µL	174.00 (130.00–240.00)	174.00 (130.00–236.00)	180.50 (129.00–266.50)	0.447
Red blood cell, m/µL	3.49 (2.98–4.08)	3.49 (3.01–4.06)	3.49 (2.96–4.40)	0.966
White blood cell, K/µL	11.80 (8.10–15.70)	11.70 (8.20–15.70)	12.35 (7.80–15.75)	0.861
Anion gap, mmol/L	13.00 (11.00–17.00)	13.00 (11.00–16.00)	16.50 (13.50–20.00)	**<0.001**
Total calcium, mmol/L	8.50 (8.00–8.90)	8.50 (8.00–8.90)	8.50 (7.80–9.15)	0.985
Chloride, mmol/L	103.00 (99.00–107.00)	104.00 (99.00–107.00)	101.00 (95.00–103.50)	**0.002**
Glucose, mg/dL	127.00 (109.00–155.00)	124.00 (109.00–152.00)	139.00 (111.50–184.00)	**0.044**
Potassium, mmol/L	4.40 (4.00–4.90)	4.40 (4.00–4.90)	4.35 (4.00–5.05)	0.283
Sodium, mmol/L	138.00 (135.00–140.00)	138.00 (135.00–140.00)	137.00 (132.00–141.50)	0.261
International normalized ratio	1.50 (1.30–1.75)	1.50 (1.30–1.75)	1.75 (1.30–2.50)	**0.007**
Prothrombin time, s	16.10 (13.80–19.05)	15.80 (13.60–19.05)	19.05 (14.50–27.10)	**0.007**
Partial thromboplastin time, s	32.90 (28.20–39.91)	32.50 (27.70–39.91)	38.70 (29.85–48.65)	**0.009**
Blood urea nitrogen, mg/dL	24.00 (16.00–37.00)	21.00 (15.00–34.00)	35.00 (24.00–60.50)	**<0.001**
Creatinine, mg/dL	1.20 (0.90–1.70)	1.10 (0.80–1.69)	1.69 (1.30–2.85)	**<0.001**
NIBP, mmHg	78.00 (69.00–91.00)	80.00 (69.00–91.00)	74.50 (64.50–91.50)	0.085
Respiratory rate, insp/min	19.00 (16.00–24.00)	19.00 (15.00–23.00)	21.00 (18.00–25.00)	**0.013**
Heart rate, bpm	85.00 (75.00–100.00)	85.00 (74.00–100.00)	85.50 (77.50–106.50)	0.5
SpO_2_, %	98.00 (95.00–100.00)	98.00 (95.00–100.00)	97.00 (93.00–100.00)	0.09
ACEI, *n* (%)				**0.005**
No	237.00 (74.76%)	190.00 (71.70%)	47.00 (90.38%)	
Yes	80.00 (25.24%)	75.00 (28.30%)	5.00 (9.62%)	
ARB, *n* (%)				0.055
No	289.00 (91.17%)	238.00 (89.81%)	51.00 (98.08%)	
Yes	28.00 (8.83%)	27.00 (10.19%)	1.00 (1.92%)	
β-blockers, *n* (%)				**<0.001**
No	105.00 (33.12%)	73.00 (27.55%)	32.00 (61.54%)	
Yes	212.00 (66.88%)	192.00 (72.45%)	20.00 (38.46%)	
Aldosterone antagonists, *n* (%)				0.061
No	257.00 (81.07%)	210.00 (79.25%)	47.00 (90.38%)	
Yes	60.00 (18.93%)	55.00 (20.75%)	5.00 (9.62%)	
Diuretics, *n* (%)				0.85
No	70.00 (22.08%)	58.00 (21.89%)	12.00 (23.08%)	
Yes	247.00 (77.92%)	207.00 (78.11%)	40.00 (76.92%)	
Inotropes, *n* (%)				**<0.001**
No	159.00 (50.16%)	144.00 (54.34%)	15.00 (28.85%)	
Yes	158.00 (49.84%)	121.00 (45.66%)	37.00 (71.15%)	
Anticoagulants, *n* (%)				0.133
No	53.00 (16.72%)	48.00 (18.11%)	5.00 (9.62%)	
Yes	264.00 (83.28%)	217.00 (81.89%)	47.00 (90.38%)	
RDW, %	14.70 (13.50–16.20)	14.50 (13.30–15.80)	16.80 (14.60–19.05)	**<0.001**
SIRI	4.18 (2.04–10.82)	3.55 (1.81–8.55)	10.92 (4.68–18.34)	**<0.001**
AISI *	7.98 (2.85–21.58)	6.55 (2.70–18.44)	17.74 (6.18–50.00)	**<0.001**
SII *	10.74 (5.14–21.56)	9.60 (4.80–20.09)	18.08 (9.27–56.02)	**<0.001**

DCM: dilated cardiomyopathy; HCM: hypertrophic cardiomyopathy; RCM: restrictive cardiomyopathy; SOFA: sequential organ failure assessment; APS III: acute physiology score III; SAPS II: simplified acute physiology score II; OASIS: Oxford acute severity of illness score; NIBP: non-invasive blood pressure; SpO_2_: oxygen saturation; ACEI: angiotensin-converting enzyme inhibitor; ARB: angiotensin II receptor blocker; RDW: red blood cell distribution width; SIRI: systemic inflammation response index; AISI: aggregate index of systemic inflammation; SII: systemic immune-inflammation index. * The AISI and SII indices were derived by computing AISI/100 and SII/100, respectively. Significant *p*-values are in bold.

**Table 2:** LASSO-Cox analysis: risk factors of mortality.

Variables	HR (95% CI)	*p*-Value
Age	1.03 (1.01–1.06)	**0.02**
Male	0.29 (0.15–0.58)	**<0.001**
Myocardial infarction	2.16 (1.03–4.53)	**0.04**
Stroke	0.25 (0.05–1.26)	0.09
SOFA	1.07 (0.93–1.22)	0.37
SAPS II	1.03 (0.99–1.06)	0.12
White blood cell	0.97 (0.92–1.03)	0.38
Anion gap	1.02 (0.96–1.09)	0.53
Total calcium	1.16 (0.84–1.59)	0.37
Chloride	0.97 (0.92–1.02)	0.20
Prothrombin time	1.02 (1–1.04)	0.09
Partial thromboplastin time	1.02 (1–1.03)	**0.01**
Urea nitrogen	1.01 (0.99–1.02)	0.33
Creatinine	1.18 (0.98–1.41)	0.08
SpO_2_	0.97 (0.91–1.05)	0.49
Sepsis	1.62 (0.69–3.79)	0.27
β-blockers	0.2 (0.1–0.39)	**<0.001**
Aldosterone antagonists	0.33 (0.12–0.9)	**0.03**
Inotropes	1.92 (0.8–4.65)	0.15
Anticoagulants	1.84 (0.58–5.92)	0.30
RDW	1.14 (1.01–1.29)	**0.03**
SII	1.01 (1–1.01)	**0.03**

SOFA: sequential organ failure assessment; SAPS II: simplified acute physiology score II; RDW: red blood cell distribution width; SII: systemic immune-inflammation index. Significant *p*-values are in bold.

**Table 3:** The association between RDW, SIRI, AISI, and SII and mortality by logistic regression analyses.

		Model 1		Model 2		Model 3	
		OR (95% CI)	*p*-Value	OR (95% CI)	*p*-Value	OR (95% CI)	*p*-Value
RDW		1.33 (1.19–1.50)	**<0.0001**	1.32 (1.18–1.50)	**<** **0.0001**	1.31 (1.10–1.60)	**0.004**
RDW (quartile)							
Q1	13.2 (12.8–13.6)	reference		reference		reference	
Q2	14.8 (14.3–15.4)	2.15 (0.84–5.93)	0.12	1.85 (0.69–5.30)	0.23	1.39 (0.38–5.32)	0.62
Q3	17.3 (16.3–19.2)	6.70 (2.95–17.29)	**<0.0001**	5.60 (2.36–15.00)	**0.0002**	4.59 (1.35–18.07)	**0.02**
*p* for trend		**<** **0.0001**		**0.0001**		**0.0118**	
SIRI		1.01 (1.00–1.03)	**0.025**	1.01 (1.00–1.02)	**0.042**	1.02 (1.00–1.03)	0.074
SIRI (quartile)							
Q1	1.41 (0.92–2.04)	reference		reference		reference	
Q2	4.18 (3.16–5.76)	1.43 (0.56–3.85)	0.46	1.63 (0.59–4.71)	0.35	0.82 (0.20–3.28)	0.78
Q3	15.97 (10.85–26.45)	5.54 (2.52–13.53)	**0.0001**	5.95 (2.51–15.71)	**0.0001**	5.29 (1.46–21.12)	**0.014**
*p* for trend		**<** **0.0001**		**<0.0001**		**0.0059**	
AISI		1.00 (1.00–1.01)	**0.008**	1.00 (1.00–1.01)	**0.014**	1.00 (1.00–1.01)	0.087
AISI (quartile)							
Q1	1.78 (1.11–2.84)	reference		reference		reference	
Q2	7.98 (5.36–10.36)	1.73 (0.70–4.55)	0.25	1.87 (0.73–5.09)	0.20	1.04 (0.29–3.75)	0.95
Q3	31.15 (21.58–60.9)	5.06 (2.30–12.41)	**0.0001**	4.79 (2.03–12.44)	**0.0006**	3.64 (1.99–14.72)	0.056
*p* for trend		**<** **0.0001**		**0.0003**		**0.038**	
SII		1.01 (1.00–1.02)	**0.0005**	1.01 (1.00–1.02)	**0.0052**	1.01 (1.00–1.02)	0.19
SII (quartile)							
Q1	4.02 (3.04–5.14)	reference		reference		reference	
Q2	10.74 (8.56–12.65)	2.54 (1.03–6.88)	0.05	2.60 (1.02–7.22)	0.05	1.60 (0.43–6.33)	0.49
Q3	31.88 (21.62–58.55)	5.33 (2.33–13.81)	**0.0002**	4.94 (2.07–13.24)	**0.0006**	3.58 (1.02–14.41)	0.056
*p* for trend		**0.0001**		**0.0004**		**0.0414**	

95% CI: 95% confidence interval. Model 1: No covariates were adjusted. Model 2: Adjusted for age, gender, weight, hypertension, cancer, type 2 diabetes, hyperlipidemia, heart failure, and myocardial infarction. Model 3: Adjusted for age, gender, weight, hypertension, cancer, type 2 diabetes, hyperlipidemia, heart failure, myocardial infarction, sepsis, ACEI, ARB, β-blockers, aldosterone antagonists, diuretics, inotropes, white blood cells, anion gap, total calcium, chloride, glucose, potassium, sodium, INR, prothrombin time, partial thromboplastin time, urea nitrogen, and creatinine. RDW: red blood cell distribution width; SIRI: systemic inflammation response index; AISI: aggregate index of systemic inflammation; SII: systemic immune-inflammation index. Significant *p*-values are in bold.

## Data Availability

The data used in the present study were obtained from the MIMIC-IV database (version 3.1), which requires credential access. Researchers may obtain the dataset by applying through PhysioNet and completing the CITI training program. To support the reproducibility of our findings, the code used for data preprocessing, statistical analysis, machine learning modeling, and Bayesian multivariate joint modeling is available from the first author (S.C., si.chen.anzhen@gmail.com) upon reasonable request.

## Supplementary Materials

The following supporting information can be downloaded at: https://www.mdpi.com/article/10.3390/diagnostics15101236/s1, Figure S1: Screening the factors affecting the mortality of cardiomyopathy patients admitted to the ICU using LASSO survival regression; Figure S2: The results of Cox PH tests; Figure S3: RCS analysis of RDW, SIRI, AISI, and SII levels for 365-day mortality in DCM and HCM patients admitted to the ICU; Figure S4: The results of subgroup interaction regression analysis; Figure S5: The results of machine learning and BMJM; Figure S6: The coefficient iteration trajectory plot of BMJM. Table S1: ICD codes for cardiomyopathy complications; Table S2: GSNs for drugs used in patients with cardiomyopathy; Table S3: Missing values for all included variables in patients with cardiomyopathy; Table S4: Hyperparameter of the nine models.
