# Supplementary material for: Integrating Dynamic Red Blood Cell Distribution Width Monitoring and β-Blocker Therapy for Mortality Prediction in Intensive Care Unit Cardiomyopathy Patients: A Bayesian Multivariate Joint Model and Machine Learning Study
Source: Diagnostics (Basel). 2025 May 14;15(10):1236. doi: 10.3390/diagnostics15101236 (PMC12109794; doi:10.3390/diagnostics15101236)
Supplement: Supplementary file 1 [file diagnostics-15-01236-s001.zip › Supplementary Figures_without track.pdf]

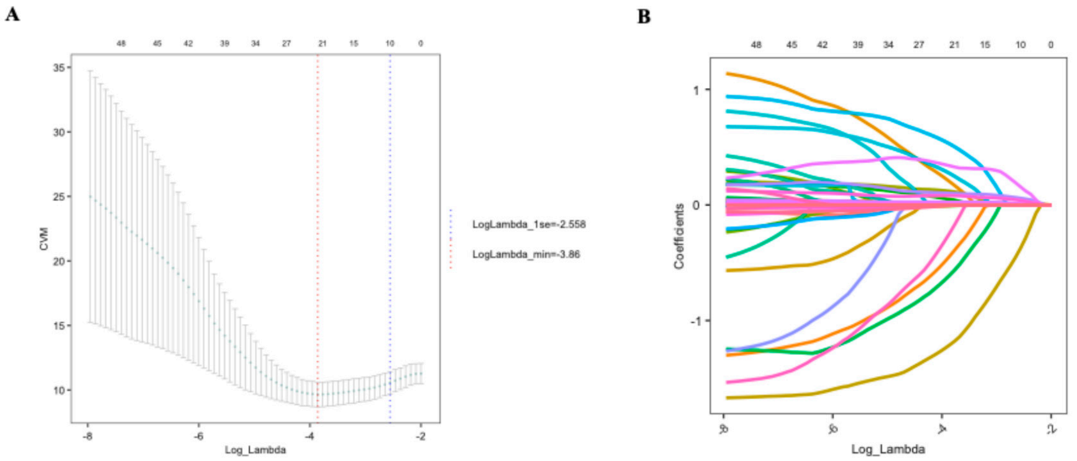

Figure S1. Screening the factors affecting the mortality of cardiomyopathy patients admitted to the ICU using LASSO survival regression. (A) Lasso regression lambda value mean square error plot; (B) Lasso regression lambda value coefficient plot.

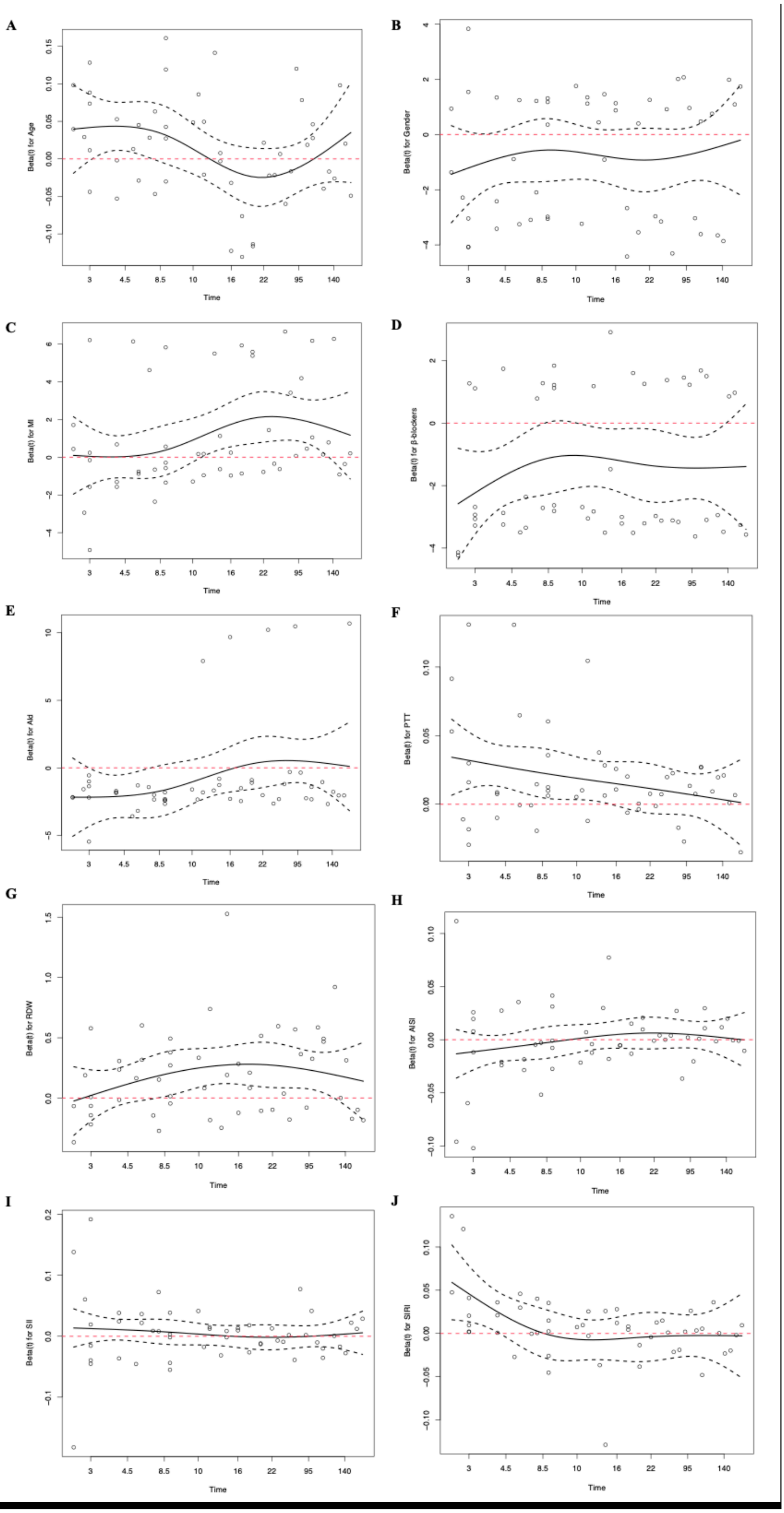

A
Beta(t) for Age
B
Beta(t) for Gender
C
Beta(t) for MI
D
Beta(t) for β-blockers
E
Beta(t) for Ald
F
Beta(t) for PTT
G
Beta(t) for RDW
H
Beta(t) for AISI
I
Beta(t) for SII
J
Beta(t) for SIRI
Time
3 4.5 8.5 10 16 22 95 140

Figure S2. The results of Cox PH tests. Abbreviations: PH: proportional hazards; RDW: red cell distribution width; MI: myocardial infarction; SII: systemic immune-inflammation index; PTT: partial thromboplastin time; Ald: Aldosterone antagonists; SIRI: systemic inflammation response index; AISI: aggregate index of systemic inflammation.

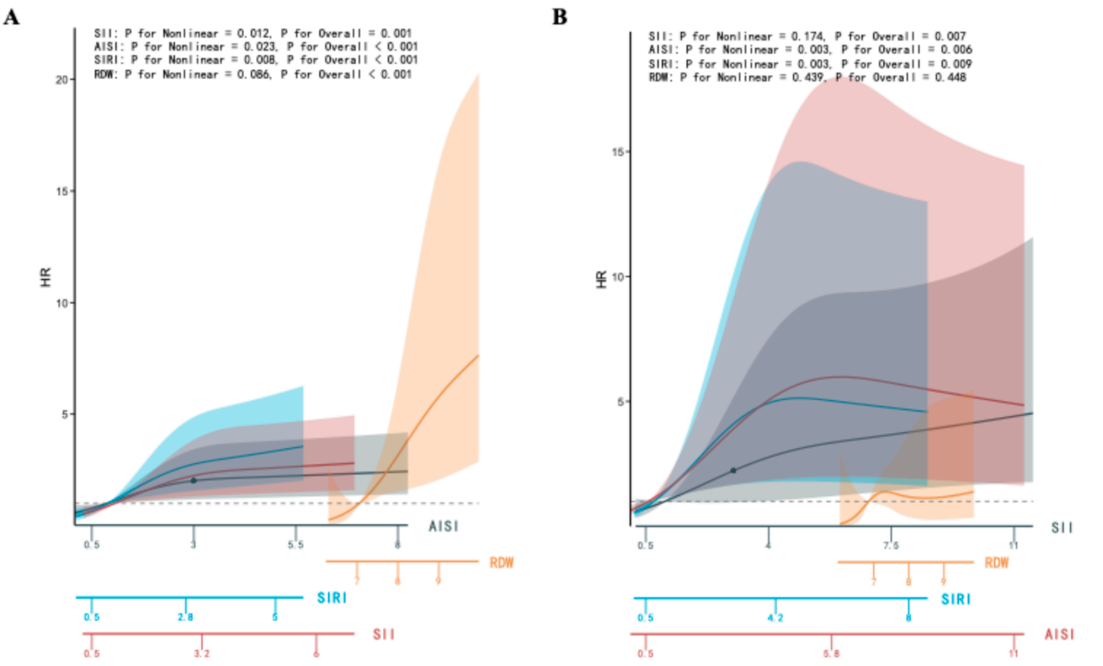

FigureS 3. RCS analysis of RDW, SIRI, AISI, and SII levels for 365-day mortality in DCM and HCM patients admitted to the ICU. (A) RCS analysis of DCM patients; (B) RCS analysis of HCM patients. RCS: Restricted cubic spline; DCM: dilated cardiomyopathy; HCM: hypertrophic cardiomyopathy; RDW: red cell distribution width; SIRI: systemic inflammation response index; AISI: aggregate index of systemic inflammation; SII: systemic immune-inflammation index; ICU: intensive care unit; HR: hazard ratios.

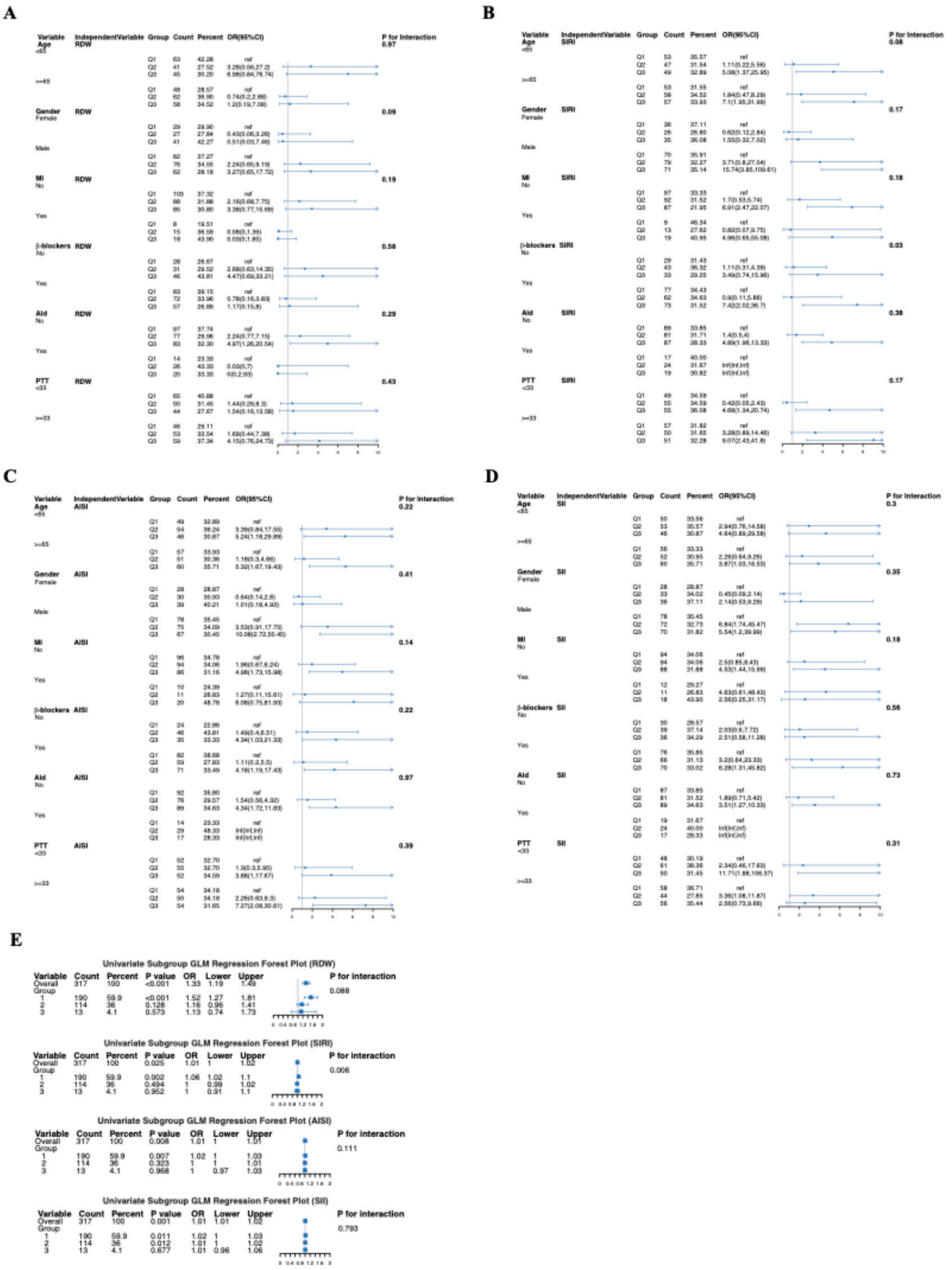

Figure S4. The results of subgroup interaction regression analysis. (A) The results of RDW subgroup interaction regression analysis; (B) The results of SIRI subgroup interaction regression analysis; (C) The results of AISI subgroup interaction regression analysis; (D) The results of SII subgroup interaction regression analysis; (E) The results of univariate subgroup GLM analysis. Abbreviations: RDW: red cell distribution width; MI: myocardial infarction; SII: systemic immune-inflammation index; PTT: partial thromboplastin time; Ald: Aldosterone antagonists; SIRI: systemic inflammation response index; AISI: aggregate index of systemic inflammation; OR: odds ratios; CI: confidence intervals; GLM: generalized linear model.

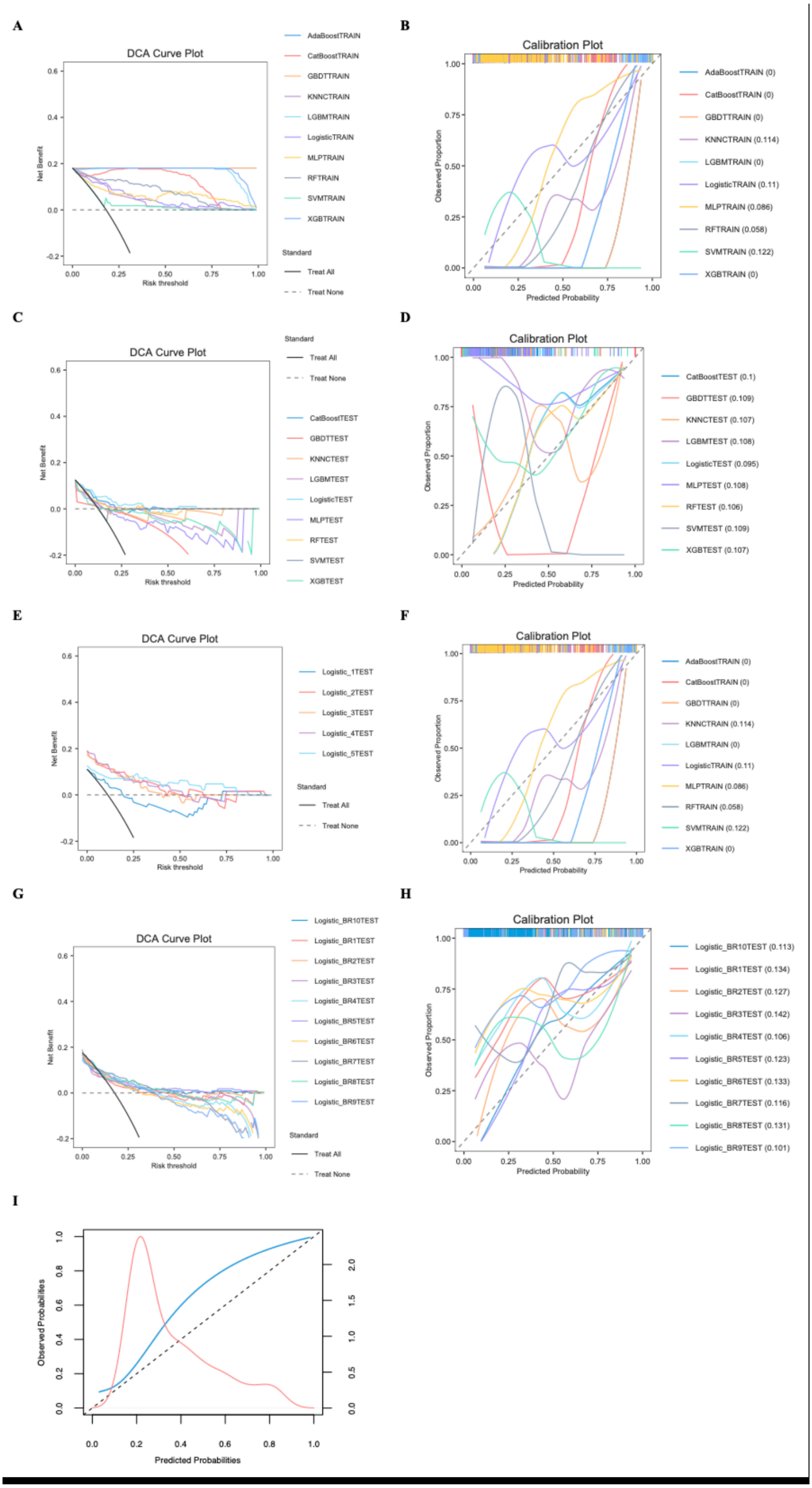
A
DCA Curve Plot
Net Benefit
Risk threshold
AdaBoostTRAIN
CatBoostTRAIN
GBDTTRAIN
KNNCTRAIN
LGBMTRAIN
LogisticTRAIN
MLPTRAIN
RFTRAIN
SVMTRAIN
XGBTRAIN
Standard
Treat All
Treat None
B
Calibration Plot
Observed Proportion
Predicted Probability
AdaBoostTRAIN (0)
CatBoostTRAIN (0)
GBDTTRAIN (0)
KNNCTRAIN (0.114)
LGBMTRAIN (0)
LogisticTRAIN (0.11)
MLPTRAIN (0.086)
RFTRAIN (0.058)
SVMTRAIN (0.122)
XGBTRAIN (0)
C
DCA Curve Plot
Net Benefit
Risk threshold
Standard
Treat All
Treat None
CatBoostTEST
GBDTTEST
KNNCTEST
LGBMTEST
LogisticTEST
MLPTEST
RFTEST
SVMTEST
XGBTEST
D
Calibration Plot
Observed Proportion
Predicted Probability
CatBoostTEST (0.1)
GBDTTEST (0.109)
KNNCTEST (0.107)
LGBMTEST (0.108)
LogisticTEST (0.095)
MLPTEST (0.108)
RFTEST (0.106)
SVMTEST (0.109)
XGBTEST (0.107)
E
DCA Curve Plot
Net Benefit
Risk threshold
Logistic_1TEST
Logistic_2TEST
Logistic_3TEST
Logistic_4TEST
Logistic_5TEST
Standard
Treat All
Treat None
F
Calibration Plot
Observed Proportion
Predicted Probability
AdaBoostTRAIN (0)
CatBoostTRAIN (0)
GBDTTRAIN (0)
KNNCTRAIN (0.114)
LGBMTRAIN (0)
LogisticTRAIN (0.11)
MLPTRAIN (0.086)
RFTRAIN (0.058)
SVMTRAIN (0.122)
XGBTRAIN (0)
G
DCA Curve Plot
Net Benefit
Risk threshold
Logistic_BR10TEST
Logistic_BR1TEST
Logistic_BR2TEST
Logistic_BR3TEST
Logistic_BR4TEST
Logistic_BR5TEST
Logistic_BR6TEST
Logistic_BR7TEST
Logistic_BR8TEST
Logistic_BR9TEST
Standard
Treat All
Treat None
H
Calibration Plot
Observed Proportion
Predicted Probability
Logistic_BR10TEST (0.113)
Logistic_BR1TEST (0.134)
Logistic_BR2TEST (0.127)
Logistic_BR3TEST (0.142)
Logistic_BR4TEST (0.106)
Logistic_BR5TEST (0.123)
Logistic_BR6TEST (0.133)
Logistic_BR7TEST (0.116)
Logistic_BR8TEST (0.131)
Logistic_BR9TEST (0.101)
I
Observed Probabilities
Predicted Probabilities

Figure S5. The results of machine learning and BMJM. (A) The DCA curves of training sets; (B) The calibration curves of training sets; (C) The DCA curves of testing sets; (D) The calibration curves of testing sets; (E) The DCA curves of cross-validation; (F) The calibration curves of cross-validation; (G) The DCA curves of bootstrap analysis; (H) The calibration curves of bootstrap analysis; (I) The calibration curve of BMJM analysis. Abbreviations: DCA: decision curve analysis; RF: random forest, XGB: extreme gradient boosting survival learner; SVM: support vector machine; BMJM: Bayesian multivariate joint model.

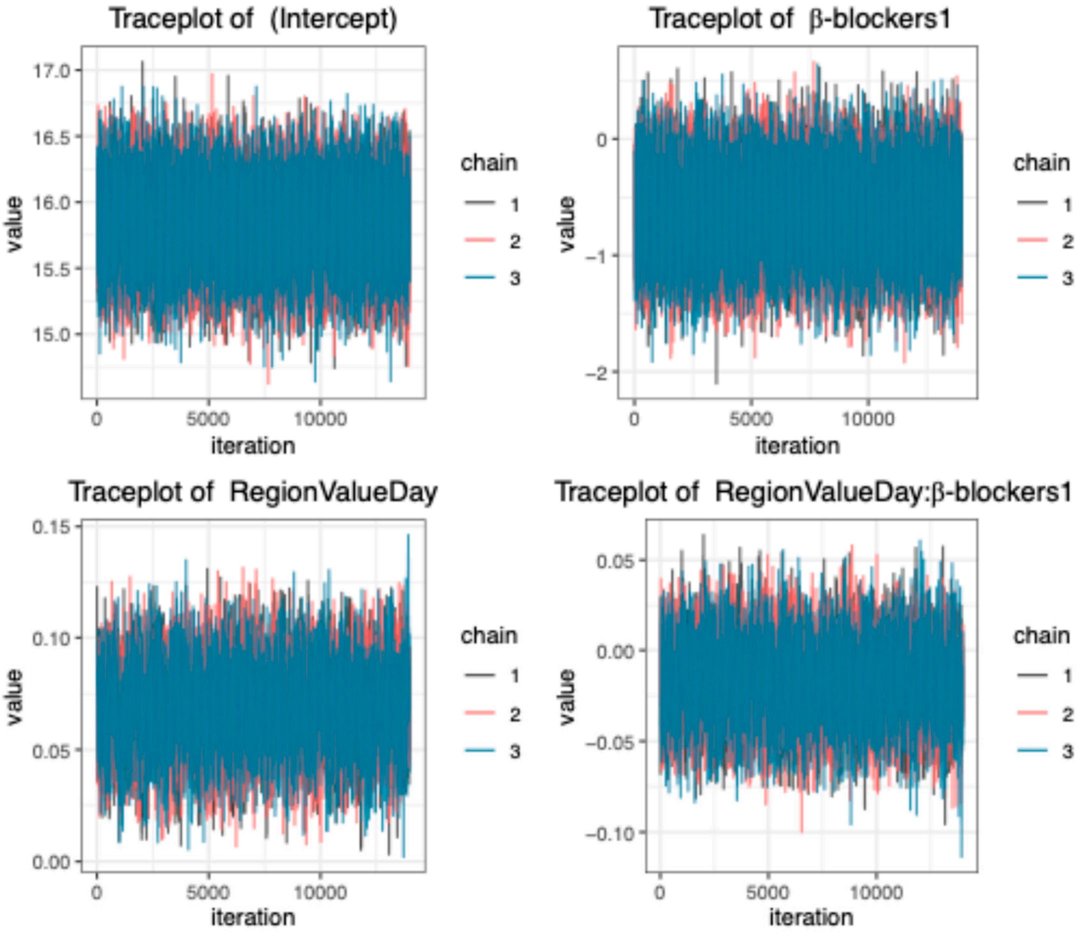

Figure S6. The coefficient iteration trajectory plot of BMJM. Abbreviations: BMJM: Bayesian multivariate joint model.
