# Supplementary material for: Integrating Dynamic Red Blood Cell Distribution Width Monitoring and β-Blocker Therapy for Mortality Prediction in Intensive Care Unit Cardiomyopathy Patients: A Bayesian Multivariate Joint Model and Machine Learning Study
Source: Diagnostics (Basel). 2025 May 14;15(10):1236. doi: 10.3390/diagnostics15101236 (PMC12109794; doi:10.3390/diagnostics15101236)
Supplement: Supplementary file 1 [file diagnostics-15-01236-s001.zip › Supplementary Tables_without track.pdf]

Table S1. ICD codes for cardiomyopathy complications

Diseases	ICD-9/10
Hypertension	4019, I10, 4011, I161, 4010
Stroke	V1254, Z8673, 431, 43820, 43811, 4359, V171, 43883, 99702, G459
Chronic kidney disease	40390, 5859, I129, N189, N183, 5853, I130, I120, 5854, N184, 5852, 40310, N182, 5855, N185, E1122
Cancer	V103, V1046, Z85828, V1083, Z853, 1985, Z8546, 1977, V1005, 1970, 1983, C787, C7951, V160, V1011, V1052, V1051, 185, Z85038, 1976, Z800, V163, 19889, Z85118, 1629, C786, C7931, Z803, C61, Z8551
Type 2 diabetes	E119, E1122, E1165, E1140, E1151, E11319, E1142, E1121, E11649, E11621, E1169, E1143, E1152, E118, E11610, E11622, E1110, E11628, E1139, E1136, 25000, 25060, 25040, 25050, 25002, 25080, 25062, 25042, 25082, 25052, 25070, 25012, 25092, 25072, 25090
Hyperlipidemia	2724, E785, E7849, E782, E784, 2722
Heart failure	4280, 42832, 42822, I5032, 42833, I5033, I5022, 42823, I5023, I509, 42830, 42843, 42831, I5030, 42821, 42842, I5021, I5020, 42820, I5031, I5043, I5042, 40491, 40291, 42841, 4289, 42840, I5084, I50810, I5041, I5082, I5040, 4281, I50814, I50811, I50813, I50812, 40201, 40492, I5089, I5083
Myocardial infarction	41000, 41001, 41002, 41010, 41011, 41012 ,41020, 41021, 41022, 41030, 41031, 41032, 41040, 41041, 41042, 41050, 41051, 41052, 41080, 41081, 41082, 41090, 41091, 41092, I21, I219, I230, I231, I232, I233, I234, I235, I236, I238, I210, I2101, I2102, I2109, I211, I2111, I2119, I2121, I2129, I213, I214, I21A1, I21A9, I222
Chronic obstructive pulmonary disease	J430, 490, 4910, 4911, 49120, 49121, 49122, 4918, 4919, 4928, 4940, 4941, 496, J40, J410, J411, J42, J431, J432, J438, J439, J44, J440, J441, J449, 4920

ICD: International Classification of Diseases.

Table S2. GSNs for drugs used in patients with cardiomyopathy

Drugs	GSNs
ACEI	000393, 000390, 000380, 017266, 000387, 000379, 015941, 026377, 016017, 016420, 015939, 023591, 018774, 016418, 026376, 018773, 016018, 016042, 016039, 024469, 000385
ARB	050289, 048401, 050805, 048400, 074408, 034468, 074410, 037015, 034469, 074409, 037017, 047126, 040910, 034470, 037016, 050290, 040659, 038686, 050288
β-blockers	050631, 005099, 019808, 005098, 005132, 028108, 028109, 075636, 023600, 015864, 052014, 005135, 005115, 005116, 005125, 048856, 017196, 005123, 005137, 063510, 005113, 005148, 005145, 063511, 036654, 051907, 043103, 064945, 005124, 005117, 005138, 005120, 068153
Aldosterone antagonists	006816, 051036, 006817, 008227, 051037, 006818
Diuretics	021408, 008209, 008205, 029832, 008163, 006816, 051036, 008151, 021409, 008210, 008213, 006817, 008166, 028915, 008227, 021718, 008217, 008201, 008223, 008182, 021406, 008148, 051037, 008183, 008221, 008224, 021410, 066298, 008206, 021407, 006818, 008149
Inotropes	062006, 064538, 066419, 004977, 064575, 064159, 066452, 004975, 004937, 000015, 028633, 023159, 065336, 000019, 004931, 064535, 000141, 004934, 017051, 021502, 004944, 004941, 004985, 000017, 004939
Anticoagulants	060301, 006562, 006549, 006561, 006532, 006543, 060304, 047325, 014198, 072661, 006560, 071450, 019331, 070414, 027994, 027993, 067642, 006522, 027995, 047021, 067862, 069699, 064219, 049808, 058354, 069570, 006541, 058355, 041433, 006544, 063997, 073295, 006542, 023446, 063998, 076985, 041434, 040868, 041662, 018076, 006559, 041432, 018073

GSNs: generic sequence numbers; ACEI: angiotensin-converting enzyme inhibitor; ARB: angiotensin II receptor blocker.

Table S3. Missing values for all included variables in patients with cardiomyopathy

Variables	Missing
Weight	0.60%
RDW	0.60%
Lymphocytes	0%
Neutrophils	0%
Monocytes	7.30%
Hematocrit	0%
Hemoglobin	0.30%
Platelet	0%
Red blood cell	0.30%
White blood cell	0%
Hemoglobin A1c	82.30%
Anion gap	0%
Total calcium	1.60%
Chloride	0.30%
Glucose	0%
Potassium	0%
Sodium	0.30%
International normalized ratio	9.80%
Prothrombin timec	9.80%
Partial thromboplastin time	10.40%
Alanine aminotransferase	27.10%
Aspartate aminotransferase	24.90%
Urea nitrogen	7.30%
Creatinine	7.30%

RDW: red cell distribution width; SIRI: systemic inflammation response index;

Table S4. Hyperparameter of the nine models.

model	hyperparameter	optimal hyperparameter
RF	Random Seed	1
	Number of Trees	100
	Loss Function	gini impurity
	Maximum Depth	3
	Minimum Samples for Split	2
	Minimum Samples for Leaf Nodes	1
	Minimum Impurity Decrease Threshold	0
XGBoost	Random Seed	1
	Weak Learner Type	gradient boosted decision tree
SVM	Random Seed	1
	Regularization Parameter	1
	Kernel Function	radial basis function
Gradient Boosting Tree	Set random seed	1
	Loss function	log_loss
	Learning rate	0.1
	Number of boosting iterations	100
	Subsample ratio	1
	Split quality evaluation method	friedman_mse
	Minimum split sample size	2
	Leaf node minimum sample size	1
	Minimum impurity decrease threshold	0
LightGBM	Set random seed	1
	Weak learner type	gbdt

	Learning rate	0.1
	Set random seed	1
	Regularization parameter	1
Logistic Classification	Penalty type selection	none
	Optimization algorithm selection	lbfgs
	Set tolerance	0.0001
	Set maximum iterations	100
	Set random seed	1
	Evaluation metric	Logloss
CatBoost	Number of iterations	100
	Tree depth	10
	Learning rate	0.1
	Set random seed	1
	Set the hidden layer structure of the perceptron	100,100,100
Multilayer Perceptron	Loss function selection	adam
	Maximum training iterations	200
	Regularization parameter	0.0001
	Initial learning rate	0.001
	Weight	uniform
k-Nearest Neighbors	Number of neighbors	5
	Neighbor algorithm	auto

RF: random forest; XGBoost: extreme gradient boosting survival learner; SVM: support vector machine.

Supplementary Table 5. Characteristics of patients with three types of cardiomyopathies.

Variable	DCM (n=190)	HCM (n=114)	RCM (n=13)	*P*-value
Age, years	64.00 (53.00 - 73.00)	67.50 (59.00 - 78.00)	73.00 (65.00 - 79.00)	***0.014***
Weight, kg	86.30 (71.40 - 103.80)	83.20 (71.90 - 97.00)	76.50 (56.53 - 107.70)	0.253
Gender				***0.002***
Female	44.00 (23.16%)	47.00 (41.23%)	6.00 (46.15%)	
Male	146.00 (76.84%)	67.00 (58.77%)	7.00 (53.85%)	
Hypertension, n (%)				***<0.001***
No	163.00 (85.79%)	76.00 (66.67%)	12.00 (92.31%)	
Yes	27.00 (14.21%)	38.00 (33.33%)	1.00 (7.69%)	
Stroke, n (%)				***0.028***
No	179.00 (94.21%)	100.00 (87.72%)	10.00 (76.92%)	
Yes	11.00 (5.79%)	14.00 (12.28%)	3.00 (23.08%)	
Chronic kidney disease, n (%)				***0.016***
No	134.00 (70.53%)	87.00 (76.32%)	5.00 (38.46%)	
Yes	56.00 (29.47%)	27.00 (23.68%)	8.00 (61.54%)	
Cancer, n (%)				0.198
No	175.00 (92.11%)	98.00 (85.96%)	11.00 (84.62%)	
Yes	15.00 (7.89%)	16.00 (14.04%)	2.00 (15.38%)	
Type 2 diabetes, n (%)				0.886
No	134.00 (70.53%)	81.00 (71.05%)	10.00 (76.92%)	
Yes	56.00 (29.47%)	33.00 (28.95%)	3.00 (23.08%)	
Hyperlipidemia, n (%)				0.589
No	91.00 (47.89%)	53.00 (46.49%)	8.00 (61.54%)	
Yes	99.00 (52.11%)	61.00 (53.51%)	5.00 (38.46%)	
Heart failure, n (%)				***<0.001***

No	16.00 (8.42%)	59.00 (51.75%)	2.00 (15.38%)	
Yes	174.00 (91.58%)	55.00 (48.25%)	11.00 (84.62%)	
Myocardial infarction, n (%)				0.279
No	163.00 (85.79%)	103.00 (90.35%)	10.00 (76.92%)	
Yes	27.00 (14.21%)	11.00 (9.65%)	3.00 (23.08%)	
Chronic obstructive pulmonary disease, n (%)				0.765
No	161.00 (84.74%)	100.00 (87.72%)	11.00 (84.62%)	
Yes	29.00 (15.26%)	14.00 (12.28%)	2.00 (15.38%)	
Hospital mortality, n (%)				0.381
No	186.00 (97.89%)	112.00 (98.25%)	12.00 (92.31%)	
Yes	4.00 (2.11%)	2.00 (1.75%)	1.00 (7.69%)	
ICU mortality, n (%)				0.498
No	159.00 (83.68%)	101.00 (88.60%)	11.00 (84.62%)	
Yes	31.00 (16.32%)	13.00 (11.40%)	2.00 (15.38%)	
LOS of hospital, day	11.45 (7.07 - 17.39)	8.96 (5.54 - 13.96)	9.58 (6.65 - 17.00)	***0.019***
LOS of ICU, day	2.68 (1.23 - 6.38)	2.30 (1.30 - 4.34)	3.81 (2.34 - 5.58)	0.169
SOFA	5.00 (3.00 - 7.00)	5.00 (3.00 - 7.00)	5.00 (5.00 - 7.00)	0.422
APS III	47.50 (36.00 - 57.00)	39.00 (29.00 - 53.00)	53.00 (45.00 - 66.00)	***<0.001***
SAPS II	35.00 (29.00 - 44.00)	36.00 (28.00 - 44.00)	44.00 (36.00 - 51.00)	0.075
OASIS	29.00 (25.00 - 35.00)	30.00 (26.00 - 34.00)	36.00 (29.00 - 41.00)	0.104
Lymphocytes, K/μL	1.38 (0.85 - 1.96)	1.31 (0.77 - 2.16)	0.87 (0.40 - 1.14)	***0.018***
Neutrophils, K/μL	9.07 (5.39 - 11.75)	9.25 (5.63 - 13.59)	8.95 (3.39 - 11.98)	0.659
Monocytes, K/μL	0.83 (0.56 - 1.18)	0.67 (0.41 - 0.96)	0.80 (0.78 - 0.92)	0.053
Hematocrit, %	33.25 (28.40 - 38.80)	30.20 (26.50 - 35.10)	32.60 (29.40 - 37.20)	***0.003***
Hemoglobin, g/dL	10.75 (9.10 - 12.40)	10.00 (8.60 - 11.20)	9.50 (8.40 - 11.80)	***0.01***
Platelet, K/μL	187.00 (135.00 - 254.00)	153.50 (115.00 - 198.00)	178.00 (152.00 - 306.00)	***0.003***

Red blood cell, m/µL	3.63 (3.05 - 4.33)	3.33 (2.85 - 3.85)	3.42 (3.34 - 3.93)	***0.019***
White blood cell, K/µL	11.65 (8.00 - 15.20)	12.45 (8.40 - 16.90)	10.50 (5.60 - 18.30)	0.445
Anion gap, mmol/L	14.00 (12.00 - 17.00)	12.00 (11.00 - 16.00)	16.00 (13.00 - 20.00)	***0.001***
Total calcium, mmol/L	8.50 (8.00 - 9.00)	8.50 (7.90 - 8.80)	8.70 (8.30 - 9.00)	0.248
Chloride, mmol/L	101.00 (98.00 - 105.00)	106.00 (102.00 - 108.00)	97.00 (94.00 - 103.00)	***<0.001***
Glucose, mg/dL	128.00 (108.00 - 161.00)	123.00 (109.00 - 149.00)	123.00 (117.00 - 137.00)	0.592
Potassium, mmol/L	4.40 (3.90 - 4.80)	4.40 (4.00 - 4.90)	4.50 (4.20 - 5.10)	0.612
Sodium, mmol/L	137.00 (134.00 - 140.00)	138.00 (136.00 - 140.00)	139.00 (138.00 - 142.00)	0.081
International normalized ratio	1.50 (1.30 - 1.80)	1.40 (1.20 - 1.75)	1.40 (1.40 - 1.75)	0.281
Prothrombin time, sec	16.30 (13.80 - 20.00)	15.65 (13.50 - 19.05)	15.50 (14.80 - 19.05)	0.317
Partial thromboplastin time, sec	33.50 (28.70 - 39.91)	31.90 (27.30 - 39.91)	30.10 (26.20 - 39.91)	0.274
Blood urea nitrogen, mg/dL	28.50 (17.00 - 42.00)	18.00 (14.00 - 30.90)	37.00 (24.00 - 84.00)	***<0.001***
Creatinine, mg/dL	1.40 (0.90 - 1.80)	1.00 (0.80 - 1.40)	2.00 (1.40 - 2.20)	***<0.001***
NIBP, mmHg	78.00 (68.00 - 91.00)	78.00 (69.00 - 91.00)	91.00 (83.00 - 104.00)	***0.012***
Respiratory rate, insp/min	20.00 (16.00 - 24.00)	18.00 (15.00 - 21.00)	20.00 (18.00 - 30.00)	***0.025***
Heart rate, bpm	86.00 (75.00 - 104.00)	83.00 (74.00 - 93.00)	81.00 (74.00 - 112.00)	0.121
SpO2, %	98.00 (94.00 - 100.00)	98.00 (95.00 - 100.00)	97.00 (94.00 - 99.00)	0.752
Ventilation, n (%)				0.747
No	42.00 (22.11%)	22.00 (19.30%)	2.00 (15.38%)	
Yes	148.00 (77.89%)	92.00 (80.70%)	11.00 (84.62%)	
Sepsis, n (%)				0.391
No	110.00 (57.89%)	65.00 (57.02%)	5.00 (38.46%)	
Yes	80.00 (42.11%)	49.00 (42.98%)	8.00 (61.54%)	
ACEI, n (%)				***<0.001***
No	123.00 (64.74%)	105.00 (92.11%)	9.00 (69.23%)	
Yes	67.00 (35.26%)	9.00 (7.89%)	4.00 (30.77%)	

ARB, n (%)				***0.004***
No	165.00 (86.84%)	111.00 (97.37%)	13.00 (100.00%)	
Yes	25.00 (13.16%)	3.00 (2.63%)	0.00 (0.00%)	
β-blockers, n (%)				0.194
No	69.00 (36.32%)	34.00 (29.82%)	2.00 (15.38%)	
Yes	121.00 (63.68%)	80.00 (70.18%)	11.00 (84.62%)	
Aldosterone antagonists, n (%)				***0.013***
No	144.00 (75.79%)	101.00 (88.60%)	12.00 (92.31%)	
Yes	46.00 (24.21%)	13.00 (11.40%)	1.00 (7.69%)	
Diuretics, n (%)				0.15
No	36.00 (18.95%)	32.00 (28.07%)	2.00 (15.38%)	
Yes	154.00 (81.05%)	82.00 (71.93%)	11.00 (84.62%)	
Inotropes, n (%)				***0.002***
No	80.00 (42.11%)	71.00 (62.28%)	8.00 (61.54%)	
Yes	110.00 (57.89%)	43.00 (37.72%)	5.00 (38.46%)	
Anticoagulants, n (%)				***0.003***
No	22.00 (11.58%)	30.00 (26.32%)	1.00 (7.69%)	
Yes	168.00 (88.42%)	84.00 (73.68%)	12.00 (92.31%)	
RDW, %	14.60 (13.70 - 16.10)	15.00 (13.20 - 16.10)	18.00 (15.70 - 19.10)	***0.012***
SIRI	4.52 (2.09 - 10.57)	3.63 (1.88 - 10.08)	8.42 (3.18 - 18.20)	0.201
AISI*	8.60 (3.39 - 22.03)	5.84 (2.42 - 15.86)	23.74 (4.83 - 50.84)	0.082
SII*	10.55 (5.47 - 23.01)	10.43 (4.74 - 19.21)	28.27 (6.11 - 51.96)	0.229

DCM: dilated cardiomyopathy; HCM: hypertrophic cardiomyopathy; RCM: restrictive cardiomyopathy; SOFA: sequential organ failure assessment; APS III: acute physiology score III; SAPS II: simplified acute physiology score II; OASIS: oxford acute severity of Illness score; NIBP: non-invasive blood pressure; SpO_2: oxygen saturation; ACEI: angiotensin-converting enzyme inhibitor; ARB: angiotensin II receptor blocker; RDW: red cell distribution width; SIRI: systemic inflammation response index; AISI: aggregate index of systemic inflammation; SII: systemic immune-

inflammation index. * The AISI and SII indices were derived by computing AISI/100 and SII/100, respectively. Significant P values are in bold.
